# Sulfolane-containing aqueous electrolyte solutions for producing efficient ampere-hour-level zinc metal battery pouch cells

**DOI:** 10.1038/s41467-023-37524-7

**Published:** 2023-04-01

**Authors:** Yu Wang, Tairan Wang, Shuyu Bu, Jiaxiong Zhu, Yanbo Wang, Rong Zhang, Hu Hong, Wenjun Zhang, Jun Fan, Chunyi Zhi

**Affiliations:** 1 Hong Kong Center for Cerebro-Cardiovascular Health Engineering (COCHE), Hong Kong SAR, Shatin, N. T 999077 China; 2 Department of Materials Science and Engineering, City University of Hong Kong, Hong Kong SAR, Kowloon 999077 China; 3 Hong Kong Institute for Advanced Study, City University of Hong Kong, Hong Kong, Kowloon 999077 China; 4 Hong Kong Institute for Clean Energy, City University of Hong Kong, Hong Kong, Kowloon 999077 China

**Keywords:** Batteries, Materials for energy and catalysis, Energy storage, Energy, Electrochemistry

## Abstract

Aqueous zinc metal batteries are appealing candidates for grid energy storage. However, the inadequate electrochemical reversibility of the zinc metal negative electrode inhibits the battery performance at the large-scale cell level. Here, we develop practical ampere-hour-scale aqueous Zn metal battery pouch cells by engineering the electrolyte solution. After identifying the proton reduction as the primary source of H_2_ evolution during Zn metal electrodeposition, we design an electrolyte solution containing reverse micelle structures where sulfolane molecules constrain water in nanodomains to hinder proton reduction. Furthermore, we develop and validate an electrochemical testing protocol to comprehensively evaluate the cell’s coulombic efficiency and zinc metal electrode cycle life. Finally, using the reverse micelle electrolyte, we assemble and test a practical ampere-hour Zn||Zn_0.25_V_2_O_5_•nH_2_O multi-layer pouch cell capable of delivering an initial energy density of 70 Wh L^−1^ (based on the volume of the cell components), capacity retention of about 80% after 390 cycles at 56 mA g^−1^_cathode_ and ~25 °C and prolonged cycling for 5 months at 56 mA g^−1^_cathode_ and ~25 °C.

## Abstract (web summary)

The negative electrode reversibility limits the lifespan of Zn metal batteries. Here, authors report an aqueous electrolyte with a reverse micelle structure that improves the reversibility of the Zn metal anode enabling the production of an ampere-hour-level pouch cell with five months lifetime.

## Introduction

Long-duration, low cost, high energy density and safe energy storage technologies are crucial for integrating intermittent renewable energy resources into future decarbonized grids^1,2^, for which aqueous Zn-ion batteries based on Zn metal (Zn^0^) anode and mildly acidic electrolytes are considered as promising candidates^3–6^. However, the lifetime of Zn-ion batteries in practical configurations and form factors like large pouch cells is limited by the severe irreversibility of Zn^0^ anode in mildly acidic electrolytes (e.g. Zinc trifluoromethanesulfonate (Zn(OTf)_2_) and ZnSO_4_ electrolyte solutions with 3 ≤ pH ≤ 5.5)^7,8^ that leads to severe H_2_ gas evolution^9^, continuous dead Zn (i.e., negative electrode regions which are electrically or ionically inert) accumulation^10^, and uncontrolled dendrite growth^6,11^.

Although intensive efforts have been devoted to resolving these irreversibility problems^5,9,12–16^, two critical issues have been significantly overlooked in this course. One is the H_2_ coevolution associated with proton (formed by water hydrolysis) reduction upon Zn^0^ electro-deposition. Ongoing efforts are focusing on developing strategies to suppress water reduction, for example, in situ/ex situ solid-electrolyte interphase (SEI) design^5,13–15^ and the highly concentrated electrolytes^9,12,13,16^. While it is well established that in mildly acidic solutions, proton reduction is more favorable than water reduction both thermodynamically and kinetically^17–19^, it was seldom studied in aqueous Zn-ion batteries. This overlooked irreversible reaction may remain unnoticeable in small-format coin cells, but would inevitably become pronounced in large-format cells^8^, such as the ampere-hour-scale pouch cells. Therefore, effectively suppressing proton reduction is more essential to realize high reversibility of Zn^0^ anode.

The other issue is that an observed high Coulombic efficiency (CE)^3,20,21^ in an asymmetric cell (e.g. Zn||Cu cell) is not necessarily indicative of a high reversibility for Zn^0^ anode in practical cells^3,22^, where the Zn^2+^ electro-reduction process usually consists of reversible Zn^0^ deposition and side reactions including H_2_ coevolution, isolated Zn (metallic state, formed by dendritic Zn^0^ losing electric connection with current collector)^23,24^, and formation of electrochemically inactive Zn-based compounds (e.g. ZnO)^5,9^. The H_2_ coevolution and formation of inactive Zn-based compounds cause permanent loss of charge while the isolated Zn may recover and contribute extra capacity in subsequent stripping processes^24^. In addition, the reversibility of Zn^0^ anode in actual battery environment significantly depends on the Zn^0^ deposition morphology^3^. Therefore, quantitatively distinguishing the contributions of each parasitic factor to the CEs and comprehensively describing the overall reversibility of Zn^0^ anode are the prerequisite to achieve high utilization of Zn^0^ in practical cells.

Here, we design and prepare an aqueous electrolyte solution where sulfolane is used to segregate waters in nanodomains by forming a “reverse micelle” core-shell architecture. The reverse micelle electrolyte (RME) is able to suppress proton reduction and improve the electrochemical plating/stripping of the Zn metal negative electrode. We also develop an electrochemical testing protocol for proper evaluation of the CE and semi-quantitatively differentiate the contribution of the parasitic reactions. Furthermore, we propose a series of reversibility criteria to describe the reversibility of zinc metal negative electrode comprehensively. Using the RME, Zn^0^ anode exhibits balanced reversibility including strong suppression on H_2_ coevolution, inhibition of dendritic and dead Zn, improved corrosion resistance, and relatively fast reaction kinetics. The improved overall reversibility enables an ampere-hour Zn||Zn_0.25_V_2_O_5_•*n*H_2_O pouch cell that demonstrates a stable long life of 390 cycles (5 months) and a competitive practical energy density of 70 Wh L_cell_^−1^ (based on the whole cell) under controlled negative/positive electrode (N/P) ratio (3.2:1), lean electrolyte (9.3 g Ah^−1^) and high areal capacity (5.5 mAh cm^−2^) at ~25 °C.

## Results

### Physicochemical and electrochemical characterizations of the reverse micelle electrolyte solution

**Fig. 1:**
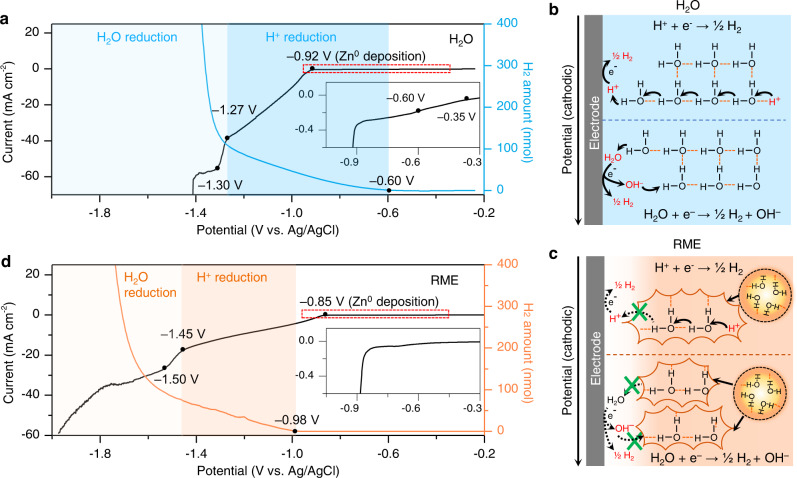
The cathodic stability of the 3 m Zn(OTf)_2_/H_2_O (H_2_O) electrolyte and the RME and corresponding proton transport mechanism. **a** Cathodic stability and H_2_ coevolution behavior for the H_2_O electrolyte determined by LSV and operando gas pressure measurement. **b** Schematic illustration of proton transport via the Grotthuss mechanism in the H_2_O electrolyte. **c** Schematic illustration of the transport limitation for protons and water in the RME. **d** Cathodic stability and H_2_ coevolution behavior for the RME determined by LSV and operando gas pressure measurement. The measurements were performed in a three-electrode cell consisting of Ti foil working electrode, activated carbon (AC) counter electrode, and Ag/AgCl reference electrode at scan rate of 1 mV s^−1^ at ~25 °C.

Using linear sweep voltammetry (LSV) combined with operando gas pressure measurement, we first examine the H_2_ coevolution behavior in the commonly used mildly acidic electrolyte 3 m Zn(OTf)_2_/H_2_O (pH=3.5, Supplementary Fig. 1). Via in situ gas chromatography (GC) we identify the major gas evolved as H_2_ (Supplementary Fig. 2). In the LSV measurements, the cathodic current starts with an exponential increase (−0.35 to −0.60 V, all the potentials in the LSV tests were normalized to the Ag/AgCl scale at 0.210 V vs. SHE) followed by a mass transport limiting section along with the H_2_ evolving from −0.60 V (Fig. 1a). This H_2_ evolution is attributed to the reduction of free protons (reaction (2))^17^ that have been generated from the hydrolysis of water by the bivalent cation Zn^2+^ (reaction (1)):

1 $${{{{{{\rm{Zn}}}}}}}^{2+}+{{{{{{\rm{nH}}}}}}}_{2}{{{{{\rm{O}}}}}}\to {[{{{{{\rm{Zn}}}}}}{({{{{{\rm{OH}}}}}})}_{{{{{{\rm{n}}}}}}}]}^{2-{{{{{\rm{n}}}}}}}+{{{{{{\rm{nH}}}}}}}^{+}$$

2 $${{{{{{\rm{H}}}}}}}^{+}+{{{{{{\rm{e}}}}}}}^{-}\to {}^{1}\!/{\!}_2{{{{{{\rm{H}}}}}}}_{2}\quad{E}^{\theta }=-0.225\,{{{{{\rm{V}}}}}}\,{{{{{\rm{vs}}}}}}.\,{{{{{\rm{Ag}}}}}}/{{{{{\rm{AgCl}}}}}}$$

3 $${H}_{2}{{{{{\rm{O}}}}}}+{e}^{-}\to {}^{1}\!/\!_2{{{{{{\rm{H}}}}}}}_{2}+{{{{{{\rm{OH}}}}}}}^{-}\quad{E}^{\theta }=-1.050\,{{{{{\rm{V}}}}}}\,{{{{{\rm{vs}}}}}}.{{{{{\rm{Ag}}}}}}/{{{{{\rm{AgCl}}}}}}$$

Such hydrolysis effect is negligible with monovalent cations such as Li^+^ or Na^+^, but becomes rather pronounced with multivalent cations, apparently induced by the strong Coulombic field of the cations^25^. The Zn^0^ deposition initiates at −0.92 V until the mass transport limitation for Zn^0^ deposition occurs at −1.30 V. From −1.27 V, both the polarization current and H_2_ evolution sharply increased, which can be attributed to the direct H_2_O reduction (reaction (3))^17^. These observations reveal that in the voltage range of Zn^0^ deposition in an electrochemical energy storage cell (e.g. overpotential <0.15 V vs. Zn^2+^/Zn), the H_2_ coevolution is dominated by the proton reduction (reaction (2))^17^.

In aqueous electrolytes, the hydrogen bonding network constitutes a matrix for fast transport of protons (H^+^) and hydroxyl anion (OH^‒^) via Grotthuss mechanism (Fig. 1b), in which the hydrogen bonds (dashed lines) and covalent bonds (solid lines) between water molecules are collectively broken and re-formed^26–28^. Therefore, controlling long-range transport of protons^29^ is an effective approach to suppress the proton reduction. Three-dimensionally constraining water into nanodomains (Fig. 1c), such as nano-sized reverse micelle, significantly slows down the transfer rate of protons via breaking the hydrogen bonding matrix and disrupting the Grotthuss proton-transport^26–28^. Here, we demonstrate that such disruption is realized when hybridizing aqueous electrolyte with a polar solvent, sulfolane, which constrains water into reverse micelle nanodomains in the resultant RME (3 m Zn(OTf)_2_/H_2_O-sulfolane (16:4, molar ratio)), thus limiting the long-range Grotthuss proton-transport and suppressing H_2_ coevolution upon Zn^0^ deposition. The sulfolane molecule consists of a strongly polar and hydrophilic group (O = S = O) sitting on a hydrophobic alkane ring. The two lone pairs of electrons on the oxygen atom (O = S = O) offer abundant hydrogen bond accepting sites that closely interact with water molecules^30^. The bulky alkane ring on the other hand would assemble itself outside of the nanoconfined water clusters. Meanwhile, as shown in Supplementary Figs. 3−6, sulfolane shows strong coordination ability to Zn^2+^ which enables the kinetics for homogeneous Zn^0^ deposition^10,12,31^ (Supplementary Note 1). To verify the effectiveness of the developed strategy for suppressing H_2_ coevolution, the cathodic stability of the RME was examined by LSV combined with operando gas pressure measurement. As shown in Fig. 1d, in contrast to the electrolyte solution using only water as a solvent (Fig. 1a), the proton reduction current was significantly reduced in the RME with H_2_ evolution initiating at −0.98 V. The Zn^0^ deposition occurs at −0.85 V until the mass transport limitation occurs at −1.50 V. The H_2_O reduction occurs at −1.45 V along with increasing of polarization current and H_2_ evolution. The presence of sulfolane simultaneously reduces the onset potential of proton reduction (from −0.60 V to −0.98 V) and water reduction (from −1.27 V to −1.45 V) in comparison to the electrolyte solution using only water as the solvent, thus confirming the ability of sulfolane to suppress H_2_ evolution in mildly acidic aqueous electrolyte. The shifted potential of proton reduction (−0.98 V) in the presence of sulfolane allows Zn^0^ plating (−0.85 V) to occur before H_2_ evolution, thus rendering the Zn^0^ deposition process more reversible. Note that the suppressed H_2_ coevolution should not be solely contributed by the slightly increased pH (4.3 vs. 3.5, Supplementary Fig. 1), given that the equilibrium potential can shift by ~0.048 V according to the Nernst equation (*E* = *E*^*θ*^ − 0.059×pH).

**Fig. 2:**
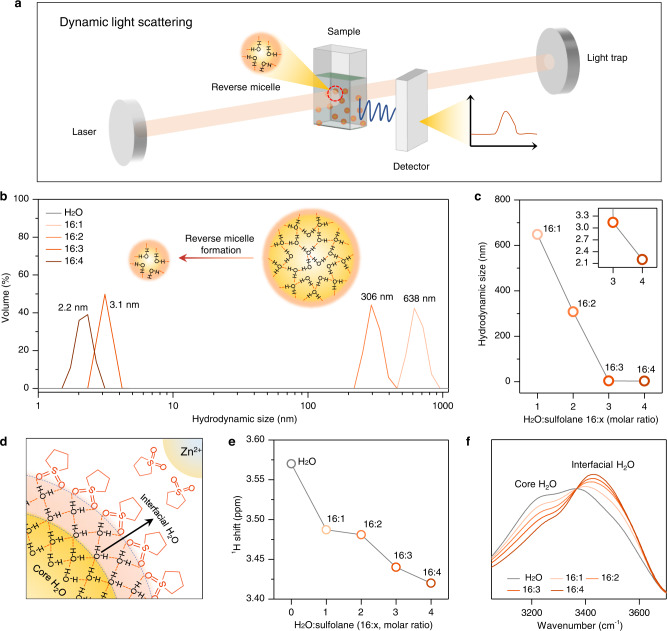
The structure of the RME and the interplay between water and sulfolane. **a** Schematic illustration of the DLS technique. **b** DLS analysis for the hydrodynamic size distribution of the water clusters in the 3 m Zn(OTf)_2_/H_2_O (H_2_O) and 3 m Zn(OTf)_2_/H_2_O-sulfolane (H_2_O:sulfolane=16:x, x = 1, 2, 3, 4, molar ratio) electrolytes. **c** The hydrodynamic size of water cluster as a function of the H_2_O:sulfolane molar ratio. **d** The schematic for the structure of the reverse micelle in the electrolyte. **e** The ^1^H NMR spectra shift for the H_2_O electrolyte and 3 m Zn(OTF)_2_/H_2_O-sulfolane (16:x, x = 1, 2, 3, 4) electrolytes. **f** Normalized FTIR spectra of the H_2_O electrolyte and 3 m Zn(OTf)_2_/H_2_O-sulfolane (16:x, x = 1, 2, 3, 4) electrolytes. The measurements were conducted at ~25 °C.

The structure of the RME and the interplay between water and sulfolane were analyzed by dynamic light scattering (DLS), nuclear magnetic resonance spectroscopy (NMR), and attenuated total reflectance Fourier transform infrared spectrometry (ATR-FTIR). The DLS technique was used to investigate the hydrodynamic size distribution of the confined water clusters in the RME (Fig. 2a). The structure of the RME was investigated by varying the H_2_O:sulfolane molar ratio from 16:0 to 16:4 with ionic conductivity ranging from 24.0 to 10.5 mS cm^−1^ (~25 °C, Supplementary Fig. 1c). As shown in Fig. 2b, the DLS analysis shows that in the electrolyte solution using only water as the solvent, no detectable water cluster was found, whereas an obvious peak with average hydrodynamic size of 638 nm was detected with the addition of sulfolane (H_2_O:sulfolane = 16:1), indicating that the bulk water has been sequestered into isolated clusters by sulfolane. Increasing the sulfolane concentration to H_2_O:sulfolane ratio of 16:3 further shrinks the main hydrodynamic radius to 3.1 nm (50% of volume). Further increasing sulfolane content (H_2_O:sulfolane = 16:4) leads to all the free water (100% of volume) sequestered into nano-sized clusters with average size of 2.2 nm which falls into the size range of “reverse micelle” (1 − 20 nm)^32^. The sharp decrease of the hydrodynamic radius size as a function of the sulfolane concentration (Fig. 2c) could be due to the preferential coordination of sulfolane to Zn^2+^ at low concentration (H_2_O:sulfolane = 16:1 and 16:2) compared with coordinating to H_2_O due to the limited amount of sulfolane and the higher stability of “Zn^2+^−sulfolane” complex which is supported by the lower solvation energy of “Zn^2+^−sulfolane” complex (e.g. −17.881 to −15.356 eV, Supplementary Fig. 4) compared with the hydrogen bond energy (−0.2 to −40 kcal mol^−1^, 1 eV = 23 kcal mol^−1^)^33^. When the coordination of Zn^2+^ tends to saturate as the increasing of sulfolane, the extra sulfolane will significantly coordinate with H_2_O and form the “reverse micelle” structure (H_2_O:sulfolane = 16:4). This behavior was supported by the similar coordination number of the sulfolane to Zn^2+^ at low concentration and high concentration (4.8 versus 5.4, Supplementary Fig. 5a–f) and the nearly identical chemical shift of the ^67^Zn signal in the NMR spectra (Supplementary Fig. 5g), which suggest the sulfolane was firstly coordinating to the Zn^2+^. It is reported that in the reverse micelles, the confined water consists of two regions of distinct shapes, *i.e*. a core–shell system^34^ (Fig. 2d), and the water molecules in the shell/interfacial region interact with the hydrophilic group of the surfactant differentiating themselves from the core water^34^. These descriptions are consistent with the results of NMR and FTIR for RME (Fig. 2e−2f). Along increasing the sulfolane concentration from H_2_O:sulfolane ratio of 16:1 to 16:4, a gradual up-field shift of the ^1^H on water molecule was observed, which was indicative of a gradually increased electron density on this nucleus because of intensified hydrogen bonding (S = O‧‧‧H − O)^29,35^ between water and sulfolane molecules (Fig. 2e and Supplementary Fig. 7). Meanwhile, the O − H stretching band (3000 − 3700 cm^−1^)^34^ in the FTIR absorption spectra (Fig. 2f) shows a rising trend in frequencies >3400 cm^−1^ and a decreasing trend in frequencies <3400 cm^−1^ as a function of the increasing of sulfolane concentration or decreasing of hydrodynamic size of water cluster (Fig. 2c). The spectra with frequencies higher than 3400 cm^−1^ was mainly contributed by the absorption of interfacial water and the spectra with frequencies lower than 3400 cm^−1^ was mainly contributed by the absorption of core water^34^. Therefore, the relative growth trend of interfacial water/core water is inversely proportional to hydrodynamic radius of the water cluster (r), *i.e*. interfacial water/core water ∝ 1/r. Moreover, a considerable blue shift from 3400 cm^−1^ (H_2_O:sulfolane = 16:1) to 3430 cm^−1^ (H_2_O:sulfolane = 16:4) was detected. Together, the experimental and calculation results indicate that upon introducing sulfolane into the aqueous electrolyte (H_2_O:sulfolane=16:1 and 16:2), the sulfolane molecules firstly coordinate with Zn^2+^ (Supplementary Fig. 5), as the coordination becomes saturate, further increasing sulfolane content (H_2_O:sulfolane=16:3) leads to the vast formation of hydrogen bond between the hydrophilic group (O = S = O) in sulfolane and the H atom in H_2_O (Fig. 2e). At the same time, the hydrophobic alkane rings on the other hand assembled themselves outside of the nano-sized water clusters (3.1 nm, Fig. 2d), thus, representing a “reverse micelle” architecture. As the H_2_O:sulfolane ratio increases to 16:4, all the free water was sequestered into “reverse micelle” nanodomains (2.2 nm) with well-shaped core water and interfacial water, as can be seen in the FTIR spectra (Fig. 2f)^34,36^. The nanoconfinement effect for water significantly hinders the molecular motions and proton delocalization, thus disrupting the transport pathway via Grotthus mechanism^26–28,37^. This mass transport limitation results in a “reactants starved” state at the electrode surface (Fig. 1c) that subsequently suppresses the proton reduction as observed in Fig. 1d.

The cathodic stability of the 2 m Zn(OTf)_2_/sulfolane electrolyte (denoted as sulfolane electrolyte) was also examined by LSV combined with operando gas pressure measurement. As show in Supplementary Fig. 8, the Zn^0^ deposition occurs at −0.83 V followed by a mass transport limitation at −1.23 V until −1.45 V where the solvent/ion decomposition could occur. Negligible gas evolution was detected in the entire cathodic scanning process, suggesting no proton/H_2_O reduction takes place. The Zn^0^ deposition current (−7.4 mA cm^−2^ at −1.23 V) is lower than that in the RME ( − 25.5 mA cm^−2^ at −1.50 V) indicating a lower mass transport/ionic conductivity (1.9 mS cm^−1^ vs. 10.5 mS cm^−1^ at ~25 °C, Supplementary Fig. 1c) and higher Zn^2+^ de-solvation energy (Supplemental Fig. 4). This is further supported by the higher charging/discharging overpotentials and inadequate cycling behavior at high rates in the sulfolane electrolyte (Supplemental Fig. 8b−f). We further conduct the LSV test combining with operando gas pressure measurement using two different organic co-solvents ethylene glycol (EG)^10,31,38^ and dimethyl sulfoxide (DMSO)^3^. Both co-solvents exhibit strong Zn^2+^ coordination ability and strong hydrogen bond-formation ability^3,10,31,38^, however, no reverse micelle structure was formed in the EG-H_2_O and DMSO-H_2_O electrolytes, as evidenced by the negligible change in the interfacial/core water population on the FTIR spectra (Supplementary Fig. 9a, b). Both the LSV curves collected from the EG-H_2_O and DMSO-H_2_O electrolytes present an obvious limiting current section (Supplementary Fig. 9c, d), which is analogous to the scenario in the electrolyte solution using only water as the solvent (Fig. 1a). The decreased proton reduction potentials of 0.05 V in the EG-H_2_O electrolyte (−0.65 V vs. −0.60 V) and 0.15 V in the DMSO-H_2_O electrolyte (−0.75 V vs. −0.60 V) are lower than that in the RME (0.38 V, − 0.98 V vs. −0.60 V). These observations confirm the role of the reverse micelle structure in suppressing proton reduction and H_2_ coevolution. In such structure, the sulfolane serves as a proton transfer inhibitor in the mildly acidic electrolyte, which is significantly different from the nonaqueous (aprotic)/aqueous (neutral) Li-ion electrolytes^30,39,40^.

### Electrochemical and physicochemical characterizations of the Zn metal electrode with reverse micelle electrolyte

The CE of asymmetric lab-scale cells, for instance metal||Cu cell, defined as the stripping capacity from the Cu electrode (*Q*_*s*_) over the plating capacity on the Cu electrode (*Q*_*p*_) (Eq. (1)), is usually used as quantitative indicator to evaluate the plating/stripping reversibility in a metal electrode^3,20,21^.

1 $${{{{{\rm{CE}}}}}}(\%)=\,\frac{{Q}_{s}}{{Q}_{p}}\times 100\%$$

**Fig. 3:**
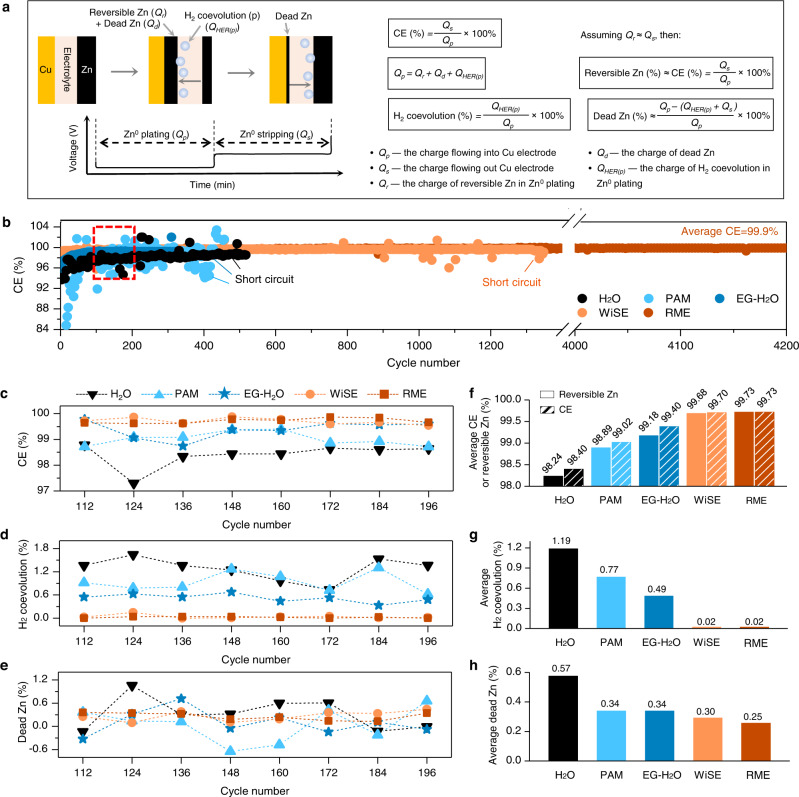
Modified CE protocol to evaluate the plating/stripping reversibility of Zn^0^ anode in the H_2_O electrolyte, the EG-H_2_O electrolyte, the PAM electrolyte, the WiSE, and the RME in extended cycles. **a** Schematic illustration of the modified CE protocol. **b** CE of Zn(50 µm)||Cu asymmetric cell in the five electrolytes. CE (**c**), contribution of H_2_ coevolution (**d**), and contribution of dead Zn (**e**) as a function of selected cycles in the five electrolytes. Average CE and contribution of reversible Zn (**f**), average contribution of H_2_ coevolution (**g**), and average contribution of dead Zn (**h**) in the five electrolytes. The galvanostatic discharging and charging tests were conducted at current density of 0.8 mA cm^–2^ with areal capacity of 0.8 mAh cm^–2^ at ~25 °C.

Differentiating and quantifying the contributions of each parasitic reaction to the CEs is crucial to accurately describe the reversibility of Zn^0^ anode. Here, we established a modified CE protocol to semi-quantitatively differentiate the contributions of reversible Zn, H_2_ coevolution, and dead Zn to CE during extended cycling by combining the CE test in Zn||Cu cell and operando gas pressure measurement (Fig. 3a). We focus on the extended cycling because the irreversibility analysis for long-term performance of Zn^0^ anode is of more relevance to the practical Zn metal batteries^23,41,42^.

Considering one completed Zn^0^ plating/stripping cycle, as illustrated in Fig. 3a, the plating capacity on Cu electrode (*Q*_*p*_) is contributed by three components: reversible Zn (*Q*_*r*_), dead Zn (*Q*_*d*_), and parasitic H_2_ coevolution (*Q*_*HER(p)*_):

2 $${Q}_{p}={Q}_{r}+{Q}_{d}+{Q}_{HER(p)}$$

Now, we assume: (i) all the anodic reactions were attributed to the oxidation of metallic Zn (Zn → 2e^−^ + Zn^2+^); (ii) the H_2_ gas detected in the prolonged cycles was mainly contributed by the H_2_ coevolution (2H^+^ + 2e^−^ → H_2_) during the cathodic processes, as no noticeable gas by corrosion reactions was detected after the first and second cycle (Supplementary Figs. 10, 11) implying that the corrosion reactions (e.g. 2H^+^ + Zn → H_2_ + Zn^2+^) is negligible in the subsequent cycles; (iii) the capacity of reversible Zn (*Q*_*r*_) approximates the stripping capacity, *Q*_*r*_ ≈ *Q*_*s*_ (although the *Q*_*r*_ is not strictly equal to *Q*_*s*_, as discussed below). Therefore, the contributions of the three components can be estimated as:

3 $${H}_{2}\,coevolution\,(\%)=\frac{{Q}_{HER(p)}}{{Q}_{p}}\times 100\%$$

4 $$Reversible\,Zn(\%)\approx CE(\%)=\frac{{Q}_{s}}{{Q}_{p}}\times 100\%$$

5 $$Dead\,Zn(\%)\approx \frac{{Q}_{p}-({Q}_{HER(p)}+{Q}_{s})}{{Q}_{p}}\times 100\%$$

We compare Zn^0^ plating/stripping reversibility in the RME with four commonly used mildly acidic/neutral electrolytes including 3 m Zn(OTf)_2_/H_2_O (H_2_O), 3 m Zn(OTf)_2_/polyacrylamide hydrogel (PAM, pH=3.5 at ~25 °C, Supplementary Fig. 11), 3 m Zn(OTf)_2_−37% H_2_O-63% ethylene glycol (weight ratio, EG-H_2_O, pH=4.1 at ~25 °C)^31^, and 2 m Zn(OTf)_2_−20 m LiTFSI in H_2_O (WiSE, pH=6.5 at ~25 °C)^9^. The ionic conductivity of the RME is 10.5 mS cm^−1^ at ~25 °C which is lower than that of the H_2_O electrolyte (24.0 mS cm^−1^ at ~25 °C) but comparable with that of the WiSE (10.7 mS cm^−1^ at ~25 °C) as shown in Supplementary Fig. 1d. This was consistent with the electrochemical impedance spectroscopy (EIS) results that the Zn||Zn cell showing an ohmic resistance of 3.6 Ω in the RME which is comparable with that of in the WiSE (3.5 Ω, Supplementary Fig. 12 and Supplementary Table 1). In addition, the charger transfer resistance of the RME (69 Ω) is lower than that of the WiSE (105 Ω) but higher than that of the H_2_O electrolyte (50 Ω, Supplementary Fig. 12). The higher charge transfer resistance of the RME than that of the H_2_O electrolyte is mainly due to the higher de-solvation energy of Zn^2+^ from the sulfolane-dominated solvation sheath as evidenced by the calculation results (Supplementary Fig. 4), which leads to the higher overpotential of Zn^0^ plating in the RME (Supplementary Fig. 13a). The tests were conducted between the 112nd and 196th cycles, where the CEs have reached a relatively stable state as indicated in Fig. 3b. The cells were cycled at 0.8 mA cm^–2^ with 0.8 mAh cm^–2^ which are standard battery testing parameters for aqueous zinc metal cells (e.g. 0.5 mA cm^–2^ with 0.5 mAh cm^–2^ and 1 mA cm^–2^ with 0.5 mAh cm^–2^)^5,43^. The profiles of the galvanostatic cycles and operando gas evolution were reported in Supplementary Figs. 13‒17. Figure 3c‒e shows the CEs and the calculated contributions of H_2_ coevolution and dead Zn according to Eqs. (1), (3), and (5) for the extended cycles.

Two scenarios occur when estimating the contribution of dead Zn according to Eq. (5): (i) *Q*_*p*_ ‒ *(Q*_*HER(p)*_ + *Q*_*s*_*) ≥* 0, *i.e*. dead Zn (%) *≥* 0; (ii) *Q*_*p*_ ‒ *(Q*_*HER(p)*_ + *Q*_*s*_*) <*0, *i.e*. dead Zn (%) <0. This is closely related to the recovery of isolated Zn in the stripping process which will be discussed in detail later. The cycles featured with “dead Zn (%) *≥* 0” were selected to estimate the average contribution of reversible Zn, H_2_ coevolution, and dead Zn as shown in Fig. 3f‒h. In Fig. 3c and Fig. 3f, the reversible Zn contributes to 99.73% and 99.68% of the deposited capacity in the RME and the WiSE in comparison with that of 98.24%, 98.89% and 99.18% in the H_2_O, the PAM and the EG-H_2_O electrolytes, respectively. In Fig. 3d and g, the H_2_ coevolution from the RME and the WiSE maintains at a relatively low level of ~0.02% which is significantly lower than that from the H_2_O (1.19%), the PAM (0.77%) and EG-H_2_O (0.49%) electrolytes. In Fig. 3e and h, the dead Zn accounts for 0.25% of the total plating capacity in the RME whereas accounts for 0.30% in the WiSE, ~0.34% in the PAM and the EG-H_2_O electrolytes, and 0.57% in the H_2_O electrolyte, respectively. These results indicate that the designed RME promotes improved plating/stripping reversibility of Zn^0^ anode with highest reversible Zn^0^ deposition, negligible H_2_ coevolution and lowest dead Zn accumulation. Such electrochemical behavior was also investigated and validated via the Aurbach CE measurement^3,21,44^ as shown in Supplementary Fig. 18.

We further evaluate three different scenarios: (i) in Fig. 3c‒e, the CE, H_2_ coevolution, and dead Zn demonstrate a relatively stable state across the selected cycles in the RME and the WiSE but present fluctuating patterns in the H_2_O, the PAM and the EG-H_2_O electrolytes; (ii) in Fig. 3e, negative values for dead Zn were found in the H_2_O electrolyte (112nd, 184th and 196th cycle), the PAM electrolyte (148th, 160th and 184th cycle), the EG-H_2_O electrolyte (112nd, 148th, 172nd and 196th cycle), and the WiSE (124th cycle); (iii) in Fig. 3f, the average reversible Zn was nearly identical to the average CE in the RME and the WiSE, but lower than the average CE in the H_2_O, the PAM and the EG-H_2_O electrolytes.

These observations are closely related to the dendrite-formation and the recovery of isolated Zn (see Supplementary Figs. 19‒21 and Supplementary Note 2 for the discussion). The dead Zn comes from two sources^23^ (it is hardly to quantitatively measure and differentiate them in the extended cycling): (i) inactive Zn (*Q*_*in*_), consisting of the electrochemical inactive species such as ZnO (existing as Zn^2+^) and is considered “permanently dead” or electrochemically inert;^5,45^ (ii) isolated Zn (*Q*_*is*_), identified as dendritic structures with loose electric contact with substrate/current collector (existing as metallic Zn or Zn^0^)^23,24^ and is considered “semi-permanently dead”. This means the isolated Zn is capable to recover and contribute extra capacity in subsequent stripping processes due to its dynamic access to the current collector that could be determined by various factors such as the “leaking” interphase and the electrochemical overpotential applied by the electric field in the electrolyte^24^ (see the discussion in Supplementary Note 2). One direct evidence for this behavior is the CEs exceeding 100% as observed in the H_2_O, the PAM, the EG-H_2_O, and the later cycles in the WiSE (Fig. 3b). Therefore, the stripping capacity (*Q*_*s*_) is contributed by two components: the reversible Zn (*Q*_*r*_) formed in the cycle considered for the calculation (major contribution) and the isolated Zn (*Q*_*is*_) accumulated in previous cycles (minor contribution), then the assumption (iii) (*Q*_*r*_ ≈ *Q*_*s*_) can be written as:

6 $${Q}_{s}={Q}_{r}+{Q}_{is}$$

And Eqs. (1), (4) and (5) can be re-written as:

7 $$CE(\%)=\frac{{Q}_{r}+{Q}_{is}}{{Q}_{p}}\times 100\%$$

8 $$Reversible\,Zn(\%)\approx CE(\%)=\frac{{Q}_{r}+{Q}_{is}}{{Q}_{p}}\times 100\%$$

9 $$Dead\,Zn(\%)\approx \frac{{Q}_{p}-\,({Q}_{HER(p)}+{Q}_{r}+{Q}_{is})}{{Q}_{p}}\times 100\%$$

Based on these knowledges, the three scenarios can be explained as following. First, for the scenario (i), the fluctuation of H_2_ coevolution in the H_2_O, the PAM, and the EG-H_2_O electrolytes (Fig. 3d) can be caused by the varying electrode surface area due to the uneven distribution of Zn dendrite^8^ (Supplementary Fig. 22a‒c). The fluctuation of CE and dead Zn (Fig. 3c and e) can be contributed by two factors: the fluctuation of H_2_ coevolution (or *Q*_*HER(p)*_), which influenced the ratio of reversible Zn (*Q*_*r*_) in the plating process (Eq. (2)) thus the fluctuation of CE (Eq. (7)) and dead Zn (Eq. (9)); and the variation of isolated Zn (*Q*_*is*_) contribution in the stripping process (Eq. (7) and Eq. (9)) due to their random distribution. Second, for the scenario (ii), when the contribution of isolated Zn (*Q*_*is*_) is high enough that leads to *Q*_*HER(p)*_ + *Q*_*r*_ + *Q*_*is*_ > *Q*_*p*_, the estimated dead Zn will become negative (Eq. (9)), which explains the negative values observed in the H_2_O, the PAM, the EG-H_2_O and the WiSE (Fig. 3e). Third, for the scenario (iii), the prevalent recovery of isolated Zn leads to the large difference between *Q*_*r*_ and *Q*_*s*_ (Eq. (6)), thus lower reversible Zn than average CE in the H_2_O, the PAM, and the EG-H_2_O electrolytes (Fig. 3f). Contrary to these electrolytes, in the RME and the WiSE, negligible dendritic Zn^0^ formation (Supplementary Fig. 22d, e) and minimal H_2_ coevolution (Supplementary Fig. 16‒17) lead to the relatively stable CEs as well as low dead Zn (Fig. 3c‒e, scenario (i)). The very low contribution of isolated Zn (*Q*_*is*_ = 0) due to negligible isolated Zn formation from dendritic Zn^0^ dissolution leads to the average contribution of reversible Zn nearly identical to the CE, as observed in Fig. 3f (scenario (iii)). These analyses indicate that the dendrite-formation was significantly suppressed in the designed RME which was also supported by the long lifetime of 8000 h (4200 cycles) for the Zn||Cu asymmetric cell (Fig. 3b and Supplementary Fig. 23).

**Fig. 4:**
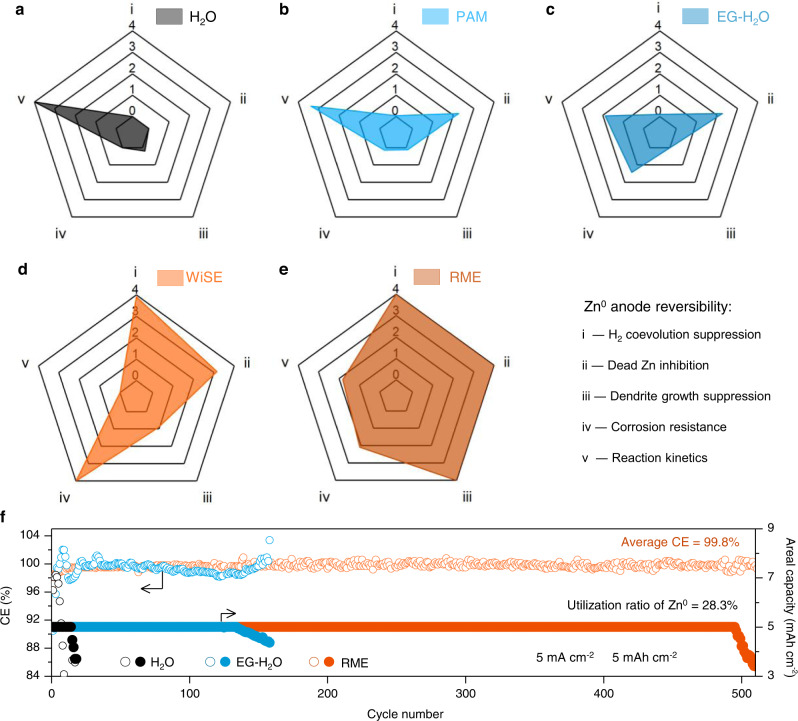
Comprehensive evaluation of Zn^0^ anode reversibility in the H_2_O, the PAM, the EG-H_2_O, the WiSE, and the RME. Radar plot assessing Zn^0^ anode reversibility in the H_2_O (**a**), the PAM (**b**), the EG-H_2_O (**c**), the WiSE (**d**) and the RME (**e**). Five normalized grades ranging from 0 to 4 are given according to the measured performance in each electrolyte. The grade of 0 represents poor H_2_ coevolution suppression, poor dead Zn inhibition, poor dendrite suppression and low corrosion resistance and low reaction kinetics; while 4 stands for the opposite, ideal properties. The calculation procedure and the grade values are provided in Supplementary Note 3 and Supplementary Table 2−4. **f** Cycling performance of the Zn(30 µm)||Cu asymmetric cell at 5 mA cm^–2^ and 5 mAh cm^–2^ with 28.3% utilization ratio of Zn^0^. The galvanostatic discharging and charging was conducted in an electrode free standing cell at ~25 °C (Supplementary Fig. 6) to minimize Zn loss on the current collector.

We further conduct a comprehensive evaluation for the overall reversibility of Zn^0^ anode taking into account five criteria: H_2_ coevolution suppression, dead Zn inhibition, dendrite growth suppression, corrosion resistance, and reaction kinetics (Fig. 4a‒e). The lifetime of the Zn||Cu cell was used to assess the dendrite growth suppression behavior (Supplementary Fig. 23). The amount of H_2_ evolved from uncycled Zn metal contacted with uncycled electrolyte was used to assess the corrosion resistance (Supplementary Fig. 11a). The anodic exchange current density was used to assess the reaction kinetics as less parasitic H_2_ coevolution would occur in anodic processes (Supplementary Fig. 24). The calculation method was included in Supplementary Note 3 and the corresponding data was listed in Supplementary Tables 2‒4. In Fig. 4a, Zn^0^ anode shows the fastest kinetics in the H_2_O electrolyte but poor reversibility in other aspects; in Fig. 4b, in the PAM electrolyte, dead Zn inhibition was improved but H_2_ coevolution, dendrite growth and corrosion effect were severe; in Fig. 4c, in the EG-H_2_O electrolyte, corrosion resistance was further enhanced, but parasitic H_2_ coevolution and dendrite growth are still severe; in Fig. 4d, in the WiSE, Zn^0^ anode shows improvement in H_2_ coevolution suppression, dead Zn inhibition, and corrosion resistance but low reaction kinetics; in Fig. 4e, in the RME, Zn^0^ anode shows balanced advantages in the five criteria. Overall, within the designed RME, Zn^0^ anode shows strong H_2_ coevolution suppression, enhanced dead Zn inhibition, dendrite suppression activity, improved corrosion resistance, and relatively fast reaction kinetics. To further confirm the dominant role of “reverse micelle” structure in suppressing the side reactions, we performed density functional theory (DFT) calculation to investigate the adsorption energy of sulfolane and EG on the Zn surface. As shown in Supplementary Fig. 25, the adsorption energy of EG ( − 1.28 eV) is closed to that of the sulfolane (−1.31 eV), however, the side reactions in the EG-H_2_O electrolyte are more serious than that in the RME. This suggests the dominant role of “reverse micelle” structure in improving the reversibility of the Zn^0^ electrode instead of molecule adsorption.

As a demonstration for the high reversibility of Zn^0^ anode in the designed RME, the Zn^0^ plating/stripping performance was examined at a high utilization ratio of 28.3% for Zn metal electrode (30 µm) and a high areal capacity of 5 mAh cm^–2^ in the Zn||Cu cell. The cell with RME sustained for 503 cycles before capacity reached to 80% (4 mAh cm^–2^) with an average CE of 99.8%. In contrast, the cell with the EG-H_2_O electrolyte sustained 160 cycles before capacity dropped to 80% and the cell with the H_2_O electrolyte only survived for 13 cycles (Fig. 4f). The premature failure of the cell with the EG-H_2_O electrolyte is consistent with the results of modified CE test that the formation of isolated Zn leads to substantial Zn loss which in turn incur capacity decay and short lifetime. Note that the WiSE and the PAM electrolyte cannot support such high current density and high areal capacity as shown in Supplementary Fig. 26. The cells were further examined at low-rate ranging from 5 mA cm^–2^ to 0.5 mA cm^–2^ with areal capacity of 5 mAh cm^–2^_,_ that the average CE reached to 99.06% in the RME at 0.5 mA cm^–2^, compared with that of 96.84% in the EG-H_2_O electrolyte and 54.50% in the H_2_O electrolyte (Supplementary Fig. 27). The high-rate capability was performed from 1 mA cm^−2^ to 10 mA cm^−2^ at 1 mAh cm^−2^, as shown in Supplemental Fig. 28. The cell with the RME shows the highest CE at various current densities with medium overpotential ranging from 90 mV (1 mA cm^−2^) to 280 mV (10 mA cm^−2^). In contrast, the poor mass transport in the WiSE leads to the premature capacity decay at current densities larger than 2 mA cm^−2^. Lower CEs were observed in the H_2_O and the PAM electrolytes. These results further confirm the improved overall reversibility of Zn^0^ anode in the designed RME.

The proposed modified CE protocol provides a way to look at some of the factors individually, which can be generally extended to examine the reversibility of other multivalent metal anodes (e.g. Al, Mg, and Mn). On the other hand, the analysis of the modified CE results reveals an underlying issue that an observed high CE is not necessarily indicative of high reversibility for Zn^0^ anode^3^. The recovery of the isolated Zn may contribute extra capacity to the stripping process leading to an artificially increased CE, but this value is not indicative of true reversibility of Zn^0^ anode, such as the scenario in the EG-H_2_O electrolyte, that the average CE was as high as ~99.7% (based on all the cycles) but the cell was shorted at ~340 cycles which resulted from the accumulation of isolated Zn. In this sense, the high CEs may not accurately reflect the reversibility of Zn^0^ electrode. Since the accumulation of isolated Zn could lead to two results: short circuit of the cell and depletion of the Zn metal, we suggest a “limited Zn^0^ reservoir” testing method (e.g. ~30% of utilization, Fig. 4f) where both the CEs and cycle life can be used as indicators to assess the reversibility of Zn^0^ anode. Highly irreversible reactions would lead to the quick depletion of the “Zn^0^ reservoir” indicated by the premature capacity decay. One of such examples is the case of the EG-H_2_O electrolyte (Fig. 4f), the formation of isolated Zn resulted in the capacity decay at only ~160 cycles. While for the electrolytes that promote high reversibility of Zn^0^ electrode, both the CE and the cycle life will be high (e.g. in the RME, the average CE = 99.8% for ~490 cycles, Fig. 4f). In other words, the “limited Zn^0^ reservoir” can highlight the irreversibility issues that are not easily discoverable with conventional CE analysis (excess amount of Zn^0^). Therefore, one can accurately estimate the reversibility of Zn^0^ electrode not only from the CE but also from the cycling life.

### Assembly and testing of Zn metal pouch cell containing the reverse micelle electrolyte solution

**Fig. 5:**
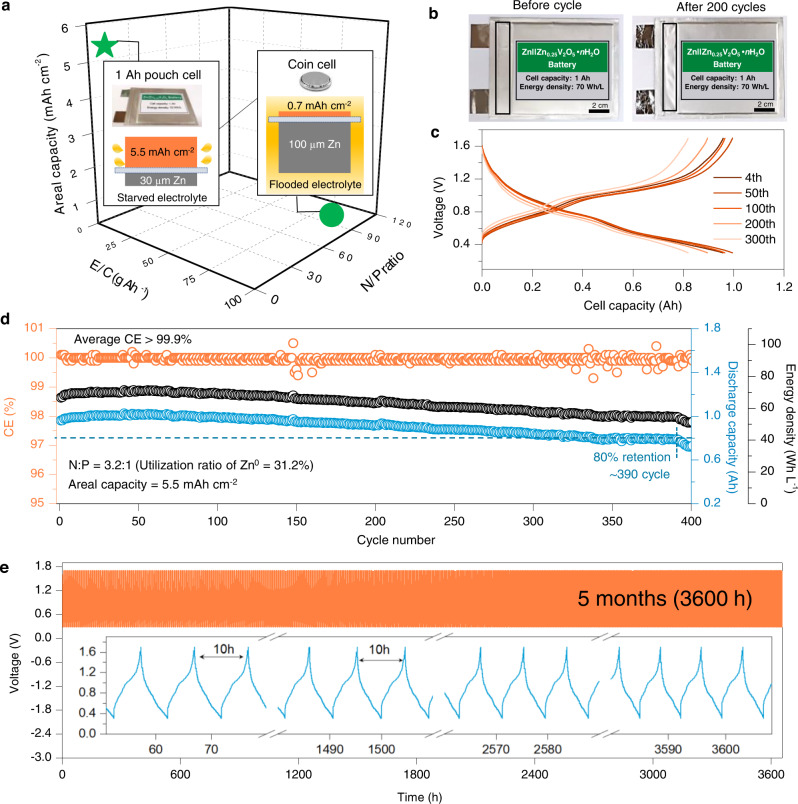
Electrochemical performance of the 1 Ah Zn||Zn_0.25_V_2_O_5_•*n*H_2_O multi-layer pouch cell using the RME. **a** Comparison of pouch cell and coin cell in terms of N/P, E/C and areal capacity. **b** The photos of the 1 Ah pouch cell before and after 200 cycles. **c** Galvanostatic charging and discharging profiles of the 1 Ah pouch cell at selected cycles at 56 mA g^−1^_cathode_ and ~25 °C.. **d** The CE, capacity, and energy density of the 1 Ah pouch cell during cycling at 56 mA g^−1^_cathode_ and ~25 °C. **e** Long-term cycling performance of the 1 Ah pouch cell at 56 mA g^−1^_cathode_ and ~25 °C. The inset shows representative voltage profiles of the pouch cell. The pouch cell was tested under an optimized external pressure of 0.1 MPa, as shown in Supplementary Fig. 29c.

Practical application for power grid energy storage requires large format batteries consisting of ampere-hour-scale large-format cells^7,46^. However, most of the reported studies rely on laboratory-scale coin cells with milliampere-hour capacity, uncontrolled Zn^0^ anode conditions, and excessive amounts of electrolytes^7,46^. For example, as shown in Fig. 5a and Supplementary Table 4, the N/P ratio can be as high as 84:1, assuming the Zn thickness of 100 µm (commonly used thickness, 58.8 mAh cm^−2^) and the cathode capacity of 0.7 mAh cm^−2^ (commonly used cathode areal capacity);^7,46^ the electrolyte/capacity (E/C) ratio can reach up to 92 g Ah^−1^, assuming the electrolyte volume of 75 µL for a CR2032 coin cell^20^. These impractical cell configuration demonstrated an prolonged but deceptive cycling stability^5^, and the energy density was significantly overestimated by excluding the excess Zn^0^ anode^5,7,8,46^. For instance, the energy density of a previously reported Zn||V_2_O_5_ coin cell^47^ is only ~1.38 Wh L^−1^_cell_ if the whole volume of the coin cell was included (see the calculation in Supplementary Table 6). Of course, this is unfair when comparing with large-format pouch cells, as in the coin cell configuration main weight and volume come from the inactive packaging materials (the coin case and cap). Nevertheless, this comparison reveals the coin cell as an impractical and limited system for fully understanding the battery cycling properties.

Here we demonstrated an ampere-hour-scale Zn||Zn_0.25_V_2_O_5_•*n*H_2_O pouch cell with strictly controlled N(30 μm, 17.6 mAh cm^−2^)/P(5.5 mAh cm^−2^) ratio of 3.2:1 and E/C ratio of 9.3 g Ah^−1^ (7 mL Ah^−1^) via using the designed RME (Fig. 5a, Supplementary Table 5 and Supplementary Figs. 29−30). The pouch cell was cycled at a low specific current of 56 mA g^−1^_cathode_ (~0.2 C, ~10 h for one cycle) and the Zn_0.25_V_2_O_5_•*n*H_2_O positive electrode was utilized to ~80% of its theoretical capacity (assuming theoretical capacity of 350 mAh g^−1^, Supplementary Fig. 31). These assembly and testing conditions are more strict than those proposed for non-aqueous Li metal pouch cells^20^. Owing to these rigorously controlled parameters, the pouch cell demonstrates an initial practical energy density of 70 Wh L^−1^_cell_ (based on the total weight of the cell components excluding the pouch bag and electrolyte volume, see Supplementary Note 4 and Supplementary Table 7), which is higher than the reported coin cell (1.38 Wh L^−1^_cell_)^47^ and comparable with that of the commercialized Lead-acid batteries (35 − 90 Wh L^−1^_cell_)^48^. The initial specific energy of the pouch cell based on the whole cell was calculated to be 31 Wh Kg_(cell)_^−1^ (Supplementary Note 5) which is also comparable with the commercialized Lead-acid batteries (10 − 40 Wh Kg^−1^)^48^.

Fig. 5b−e show the package swelling behaviors before and after 200 cycles and the cycling performance of the 1 Ah Zn||Zn_0.25_V_2_O_5_•*n*H_2_O pouch cell using the RME. The cycle life, defined as the cycle number upon reaching 80% capacity retention, is as long as 390 cycles (1.1 mA cm^−2^), which translates to a lifetime of 5 months (3600 h), as demonstrated in Fig. 5d−e. This cycling stability of 3600 h dramatically outperforms the previously reported Zn-ion pouch cells, e.g. 223 h for a Zn||MnO_2_ pouch cell^49^ (Supplementary Table 8). The cyclability improvement by controlling proton activity is consistent with a previous study^50^. A high CE of greater than 99.9% was delivered, confirming the prolonged cyclability with minimal loss of active materials and electrolytes. The high stability between the RME and the cell components upon cycling was further verified from four aspects. First, as shown in Fig. 5b, compared with the pristine packing, no significant swelling due to H_2_ gas evolution was observed after 200 cycles, confirming the effective H_2_ gas suppression ability of the RME. Second, in Fig. 5c, minimal voltage polarization was observed before 200 cycles, indicating a negligible accumulation of passivated species (e.g. Zn(OH)_2_ and ZnO) and minimal loss of electrolyte because of H_2_ releasing. Third, in Supplementary Fig. 32, the self-discharge test of a Zn||Zn_0.25_V_2_O_5_•*n*H_2_O pouch cell with N(10 µm, 5.9 mAh cm^−2^)/P(5.5 mAh cm^−2^) ratio of 1.07:1 shows that after resting for 60 h and 610 h at open circuit voltage (OCV) and 100% state of the charge (SOC, charged to 1.7 V), the cell can still deliver 97.9% and 78.0% of the charged capacity, respectively. The low self-discharge rate with 22.0% of capacity decay is comparable with the nickel metal hydride batteries (20–30% per month)^51^. Notably, even at high temperature of 55 °C, the cell can still sustain 82.5% of charged capacity after resting for 48 h with 100% SOC at OCV (Supplementary Fig. 33), further demonstrating the improved stability of the cell components within the RME. Fourth, the EIS results of the pouch cell show that the ohmic resistance (R_S_), the charge transfer resistance of the negative electrode (R_CT-n_), the interphase resistance of the negative electrode (R_int-n_), the charge transfer resistance of the positive electrode (R_CT-p_) and the Warburg coefficient of the Zn^2+^ in the electrode materials^52^ all become nearly constant after 10 cycles, indicating the fast construction of the cell stability at the first 10 cycles (Supplementary Fig. 34 and Supplementary Table 9). These results indicate that the designed RME successfully improves the overall reversibility of Zn^0^ anode, thereby enables its practical use in ampere-hour-scale pouch cell with long-term stability and competitive practical energy density.

## Discussion

We designed a reverse micelle electrolyte using sulfolane to constrain water in reverse micelle nanodomains, which hinders Grotthuss proton-transport pathway, thereby effectively suppresses proton reduction and improves the overall reversibility of Zn^0^ anode. To rigorously evaluate the Zn^0^ reversibility amid the varying parasitic reactions, we established a modified CE protocol that semi-quantitatively distinguishes the distribution of each parasitic factor to the overall reversibility of Zn^0^. Within the developed RME, Zn^0^ anode exhibits balanced reversibility including strong H_2_ coevolution suppression, enhanced dead Zn inhibition, dendrite suppression, improved corrosion resistance, and relatively fast reaction kinetics. The improved overall reversibility enables an ampere-hour Zn||Zn_0.25_V_2_O_5_•*n*H_2_O pouch cell to demonstrate a stable long life of 5 months (3600 h) and a competitive practical energy density under controlled negative/positive electrode (N/P) ratio (3.2:1), lean electrolyte (9.3 g Ah^−1^) and high areal capacity (5.5 mAh cm^−2^). Our established CE protocol and evaluation criteria can be extended to examine the reversibility of other polyvalent metal anode (e.g. Al, Mg, and Mn), while the reverse micelle structure opens a sustainable pathway of controlling the ion transport as well balancing the parasitic reactions in aqueous electrolytes for emerging battery chemistries.

## Methods

### Electrolytes preparation

All the salts and solvents used in the electrolytes were bought from Aladdin and used without further purification. The deionized water was purified on a MilliQ device from Millipore. The RME was prepared by dissolving 3 m Zn(OTf)_2_ (98%, Aladdin) into H_2_O:sulfolane (>99.5%, Aladdin) with molar ratio of 16:4 (or weight ratio of 37:63). The 3 m Zn(OTf)_2_−37% H_2_O-63% EG (weight content) electrolyte was prepared by dissolving 3 m Zn(OTf)_2_ into H_2_O:EG (99.8%, Aladdin) with weight ratio of 37:63. The 3 m Zn(OTf)_2_−37% H_2_O-63% DMSO (weight content) electrolyte was prepared by dissolving 3 m Zn(OTf)_2_ into H_2_O:DMSO ( > 99.9%, Aladdin) with weight ratio of 37:63. The WiSE (2 m Zn(OTf)_2_−20 m LiTFSI in H_2_O) electrolyte was prepared by dissolving 2 m Zn(OTf)_2_ and 20 m LiTFSI (>99.9%, Aladdin) into deionized water. The PAM hydrogel was synthesized as following. 2 g acrylamide was dissolved in 2 mL H_2_O under vigorously magnetic stirring at 40 °C. Then 2 mg N, N’-methylenebisacrylamide (99.9%, Aladdin) as crosslinkers and 5 mg potassium persulfate (99.5%, Aladdin) as initiator were added into above solution and maintain at 40 °C for 30 min. The mixture solution was degassed and sealed under nitrogen to remove dissolved oxygen. After that, the free-radical polymerization was carried out at 65 °C for 30 min. After drying, the 3 m Zn(OTf)_2_/PAM (PAM) was prepared by immersing the PAM in 3 m Zn(OTf)_2_/H_2_O for 8 h and wiping the liquid on the surface before use.

### Electrodes fabrications

Activated carbon (AC) electrode. The AC powder (>95%, YP50F, particle size 4 − 7 µm) was mixed with polyvinylidene difluoride (PVDF, > 99.5%, HSV900, Arkema) in N-methylpyrrolidone (NMP, 99.5%, Aladdin) solvent with weight ratio of AC:PVDF of 9:1. The obtained slurry was uniformly spread onto a petri dish and dried at 80 °C for 12 h. The obtained electrode sheets were punched out to electrodes with diameter of 12 mm and AC loading of 40–50 mg cm^–2^.

Zn_0.25_V_2_O_5_•*n*H_2_O positive electrode. Zn_0.25_V_2_O_5_•*n*H_2_O nanobelts (Supplementary Fig. 31) were synthesized according to a previous study^4^. Specifically, 2 mmol V_2_O_5_ (99.99%, Alfa Aesar) was dispersed in 50 ml of 15:1 water/acetone (volume ratio, the purity of acetone is 99.5%) mixture with 1.3 mmol of zinc acetate (99.99%, Aladdin) and transferred to a sealed Teflon vessel and maintained at 200 °C for 72 h. The obtained product was washed with distilled water and isopropanol (>99.5%), and dried at 60 °C for 24 h. To prepare the Zn_0.25_V_2_O_5_•*n*H_2_O positive electrode, the obtained Zn_0.25_V_2_O_5_•*n*H_2_O nanobelts were mixed with Super P (99.9%, TIMCAL, particle size 40 − 50 nm) and PVDF in NMP with weight ratio of Zn_0.25_V_2_O_5_•*n*H_2_O:Super P:PVDF of 6:3:1. The slurry was carefully ground in an agate mortar by hand in the open air to get totally uniform slurry and then spread onto a Ti mesh (100 mesh, 99.5%, Haifu Mesh) which was fixed in a thickness/length/width strictly controlled in-house developed electrode mode (Supplementary Fig. 30a). To get a uniform surface, the electrode was dried under −50 kPa at 60 °C for 72 h. After drying, the electrode sheet was cut to get a length of 100 mm, width of 90 mm. Then the electrode was pressed by a rolling machine (MSK 2150, MTI) to control the thickness of 260 µm (Supplementary Fig. 30b). The loading mass of Zn_0.25_V_2_O_5_•*n*H_2_O was ~20 mg cm^–2^.

### Pouch cell assembling

The pouch cell was constructed with a bipolar structure (Supplementary Fig. 29a) with two Zn foil (30 µm, >99.999%, Yudingda Metal) clamped in the middle sharing one Ti foil current collector (>99.99%, Yudingda Metal) and two pieces of Zn_0.25_V_2_O_5_•*n*H_2_O electrode on both sides separated by separators (glass fiber (Whatman), the thickness is 200 µm, the average pore diameter is 1.6 µm) and hydrophilic Celgard (the thickness is 30 µm, the average porosity is 55%). The parameter of the components is listed in Supplementary Table 7. Before assembly, the Zn_0.25_V_2_O_5_•*n*H_2_O electrodes were firstly processed by charging to 1.7 V to fully release the stored Zn^2**+**^. Here the Zn foil (9 cm × 10 cm × 30 µm) was used as the counter electrode, the RME was used as electrolyte (10 mL), a 10 cm × 11 cm × 0.2 cm Al packaging bag was used to accommodate the cell. The two positive electrode tabs and the tabs with the packaging films were bonded by the tab film (MTI) via hot-pressing. The sealing procedure was conducted by the automated vacuum sealing machine (Wuhan Geruisi New Energy Co., Ltd). All the assembling procedures were completed in atmosphere at room temperature (~25 °C). Before the cycling test, the assembled pouch cell was cycled for 5 times at 56 mA g_cathode_^−1^ to fully wet the thick electrodes as well as release the H_2_ gas caused by corrosion reactions via opening the packing film. And then the cell was re-sealed by the automated vacuum sealing machine and conducted the cycling tests.

### Electrochemical measurement

Cathodic stability tests and operando gas pressure measurement. The cathodic stability tests were performed in a three-electrode cell (MTI) consisting of Ti foil working electrode, AC counter electrode, and Ag/AgCl reference electrode (CHI). The cell was connected to the gas pressure sensor (Star sensor manufacturing Co., Ltd). Before the tests, the cell was filled with Ar gas to replace the air. The gas pressure (kPa) was translated to the gas amount (nmol) according to the ideal gas law. The LSV tests were performed on electrochemical working station (CHI 760E) at 1 mV s^−1^ at ~25 °C. To minimize the potential influence of other factors to the H_2_ evolution reaction, such as the SEI and/or the chemical reaction between deposited Zn and electrolyte, the Ti foil was firstly processed in the RME by cyclic voltammetry (CV) between −1.0 V (vs. Ag/AgCl) and −0.2 V (vs. Ag/AgCl) for one cycle (first negative scan and then positive scan). And then the Ti foil was extracted from the cell and washed by deionized water and dried in the air. Then the processed Ti foil was assembled with fresh electrolytes (the H_2_O, the RME, the EG-H_2_O, and the DMSO-H_2_O electrolyte) to conduct the cathodic stability tests.

Galvanostatic charge and discharge tests. The galvanostatic cycling of Zn||Zn (Supplementary Fig. 3a) and Zn||Cu (Fig. 3b and Fig. 4f) cells were performed in an in-house developed electrode free standing cell (Supplementary Fig. 6). The electrode fee standing cell enables the electrode area identical to current collector avoiding the Zn spreading on current collector upon deposition thus minimize the Zn loss on current collector. The contact area between stainless-steel negative current collector (304, MTI) and Zn foil (>99.999%, Yudingda Metal) was wrapped by a piece of conductive Cu tape (99.99%, MTI) to minimize the side reactions between electrolyte and stainless-steel current collector. A piece of Ti foil was placed between the copper tape and Zn foil to minimize the side reactions between Zn foil and Cu tape. The glass fiber (Φ12 mm, thickness 260 µm, Whatman) was used as separator and immersed with 30 µL electrolytes (for the H_2_O, the EG-H_2_O, the WiSE and the RME). The PAM with diameter of 9 mm, thickness of 0.5 mm was directly used without additional separator. The galvanostatic cycling was performed on a LAND Battery Testing System (Wuhan Land Electronic).

Modified CE tests. The Zn||Cu test combining operando gas pressure measurement (Fig. 3c, Supplementary Fig. 13−17) was performed in a two-electrode cell (MTI) with Cu foil as working electrode (Φ9 mm, >98%), Zn foil (50 µm, Φ9 mm, >99.999%) as counter and reference electrode. The glass fiber (Φ12 mm, thickness 260 µm, Whatman) was used as separator and immersed with 30 µL electrolyte (for the H_2_O, the EG-H_2_O, the WiSE and the RME). The PAM with diameter of 9 mm, thickness of 0.5 mm was directly used without additional separator. The galvanostatic cycling was performed on a LAND Battery Testing System (Wuhan Land Electronic).

Corrosion tests. The corrosion tests were performed in the same cell used for the modified CE tests. The H_2_ gas pressure was measured until reaching to a relative stable value where the corrosion reactions became minimized (Supplementary Fig. 10). The amount of H_2_ evolved from pristine state was used to assess the corrosion resistance of each electrolyte (Supplementary Fig. 11a).

Reaction kinetics. The Tafel plots (Supplementary Fig. 24a, b) were collected in an in-house developed three-electrode cell (Supplementary Fig. 24c) consisting of Zn foil working electrode, Zn foil counter electrode, and Ag/AgCl reference electrode (CHI 760E). The exchange current densities were calculated by fitting Tafel plots in the voltage range from −30 mV to 30 mV vs. Zn^2+^/Zn. The anodic exchange currents were used to assess reaction kinetics because of the less H_2_ coevolution in the anodic process. The tests were repeated for ~5 times until the plots became relative stable.

In situ GC measurement. The in situ GC measurement was performed on a gas chromatography system (GC 2060, Shanghai Ruimin Instruments Co., Ltd.) which was connected to the assembled electrochemical cell. The H_2_ signal was calibrated by a standard gas mixture of H_2_ (50 ppm), O_2_ (1000 ppm), CO (50 ppm), CH_4_ (100 ppm), C_2_H_6_ (50 ppm), C_2_H_4_ (50 ppm), and C_2_H_2_ (50 ppm) balanced by N_2_ (Arkonic Gases & Chemicals Inc., Hong Kong).

Electrochemical impedance spectroscopy (EIS) measurements. The cells firstly rested for 2 h until the open circuit voltage becomes stable. The EIS tests were carried out under the open circuit voltage condition at a frequency window ranging from 100 kHz to 0.1 Hz (six data points per decade of frequency) with an amplitude of 10 mV using the CHI electrochemical working station. The collected data was fitted by the EC-Lab software. The parameter of χ^2^ gives an estimation of the distance between the raw data and the fitted data and defined as:

$${{{{{{\rm{\chi }}}}}}}^{2}=\,\mathop{\sum }\limits_{i=1}^{n}\frac{{{{{{{\rm{|}}}}}}Z}_{{meas}}\left(i\right)-{Z}_{{simul}}{{{{{{\rm{|}}}}}}}^{2}}{{\sigma }_{i}^{2}}$$

Where, *Z*_*meas*_*(i)* is the measured impedance at the f_i_ frequency; *Z*_*simul*_ is a function of the chosen model; *σ*_*i*_ is the standard deviation. The parameter of χ/N^0.5^ was used to evaluate the error between raw and fitted data where N is the number of points.

In situ optical microscopy measurement. The in situ optical microscopy measurement was performed in an in-house developed optical microscopy cell (Supplementary Fig. 20) with Zn foil (50 µm) as the negative electrode, Zn_0.25_V_2_O_5_•*n*H_2_O as the positive electrode, and Zn island mimicking the isolated Zn. The Zn island was prepared by depositing Zn metal on Cu foil (5 mAh cm^−2^) and then cut into a strip with width of ~200 µm (Supplementary Fig. 20). Before the test, the cell was filled with 3 m Zn(OTf)_2_/H_2_O electrolyte and rested for 4 h to fully release H_2_ gas caused by the corrosion reactions. Then the cell was connected to electrochemical working station (CHI) and discharging at a constant current of 300 µA. The optical microscope (ZEISS Primotech) was used to in situ detect the morphology change of Zn island. At least three cells were tested for each single electrochemical experiment for ensuring the reproducibility of the results.

### Characterization

The FTIR spectra were collected from PerkinElmer Spectrum II FT-IR Spectrometer. The ^1^H NMR spectra were acquired on a Bruker AVANCE III HD 400 NMR spectrometer using deuterated DMSO as the field frequency lock. The ^67^Zn NMR spectra were acquired on a Bruker AV 600 NMR spectrometer using deuterated DMSO as the field frequency lock. The SEM images were collected from Hitachi SU4800. The ex situ SEM samples were obtained by disassembling the cell, extracting the electrode, washing by DI water for 3 times, and drying at vacuum (~25 °C) for 8 h.The DLS measurement was performed on DLS instrument (Zetasizer Nano ZS, Malvern Instruments Ltd) at 25 °C with scattering angle of 90° and laser wavelength of 632.8 nm. The ionic conductivities of the electrolytes were measured on a conductivity meter (DDS-11A, INESA) at ~25 °C.

### DFT-MD simulation

All the full-atom molecular dynamic simulations were performed on the LAMMPS software^53^. The water molecule was represented by the SPC/E model^54^. The force field parameters of Zn^2+^, OTf^–^, and sulfolane are obtained from the literature^55–57^. All the simulations were pre-equilibrated for 2 ns in the canonical ensemble and 2 ns in the isothermal-isobaric ensemble. The atomic trajectory for analysis was generated by another 2 ns production run in the isothermal-isobaric ensemble at 300 K. The time step in all simulations was set to 0.5 fs. The Coulombic interactions were computed using the pppm method. The geometric mix rule was used for non-bond interaction. The DFT calculations for sulfolane solvation energy were performed on Gaussian09 RevE^58^. The initial structures of solvent cluster were built according to the MD simulation results. The structure optimization and energy calculation were both in 6-311 + G** level, D3 Grimme’s dispersion with Becke-Johnson damping was used to describe the dispersion^59^. All cluster structures and isopycnic surface were displayed by VMD software^60^.

The adsorption energy of the sulfolane and the EG molecule on the Zn surface was performed by the Vienna Ab initio Simulation Package (VASP)^61,62^. For all calculations, the DFT-D3 dispersion correction was used^63^. In all calculations, the 2s^2^2p^2^, 1s^1^, 3s^2^3p^4^, 2s^2^2p^4^ and 3d^10^4s^2^ electrons were considered as valence electron for C, H, S, O, Zn element in used pseudopotential. The self-consistent field electronic calculation criterion was set to 10^−6^ eV. The force-based criterion of structural optimization was set to 0.02 eV Å^−1^. The cell was composed of 5×5×3 layers of Zn atoms with the (0001) surface exposing to vacuum in z-direction. The vacuum thickness is larger than 15 angstroms to prevent periodic reaction in z-direction. Then the adsorbates were put on the Zn (0001) surface and the bottom layer of Zn surface was frozen to mimic a bulk phase circumstance. A 520 eV cutoff energy and 0.25 Å^−1^ k-point mesh resolution were used for the calculation involving Zn surface. The isolated adsorbates were optimized in the box size of 1.5×1.5×1.5 nm^3^ with a single gamma k-point. The adsorption energy is calculated via:

$${E}_{{ads}}={E}_{{Zn}+{mol}}-{E}_{{Zn}}-{E}_{{mol}}$$

where the ${E}_{{Zn}+{mol}}$, the ${E}_{{Zn}}$, and the ${E}_{{mol}}$ represent the total energy of adsorbates on the Zn surface, the energy of Zn surface, and the energy of the isolated adsorbates, respectively.

## Supplementary information

Supplementary Information

## Data Availability

The data that support the findings of this study are available within the text including the Methods, and Supplementary information. Raw datasets related to the current work are available from the corresponding author on reasonable request.
