# Supplementary material for: Sulfolane-containing aqueous electrolyte solutions for producing efficient ampere-hour-level zinc metal battery pouch cells
Source: Nat Commun. 2023 Apr 1;14:1828. doi: 10.1038/s41467-023-37524-7 (PMC10067964; doi:10.1038/s41467-023-37524-7)
Supplement: Supplementary file 1 — Supplementary Information [file 41467_2023_37524_MOESM1_ESM.pdf]

Supplementary Information

Sulfolane-containing aqueous electrolyte solutions for producing efficient ampere-hour-level zinc metal battery pouch cells

Yu Wang[1,2], Tairan Wang[2], Shuyu Bu[2], Jiaxiong Zhu[2], Yanbo Wang[2], Rong Zhang[2], Hu Hong[2], Wenjun Zhang[2], Jun Fan[2]*, and Chunyi Zhi[1,2,3,4]*

[1]Hong Kong Center for Cerebro-Cardiovascular Health Engineering (COCHE), Shatin N. T. 999077, Hong Kong SAR, China
[2]Department of Materials Science and Engineering, City University of Hong Kong, Kowloon, 999077, Hong Kong SAR, China
[3]Hong Kong Institute for Advanced Study, City University of Hong Kong, Kowloon, Hong Kong, 999077, China
[4]Hong Kong Institute for Clean Energy, City University of Hong Kong, Kowloon 999077, Hong Kong

*E-mail: junfan@cityu.edu.hk; cy.zhi@cityu.edu.hk

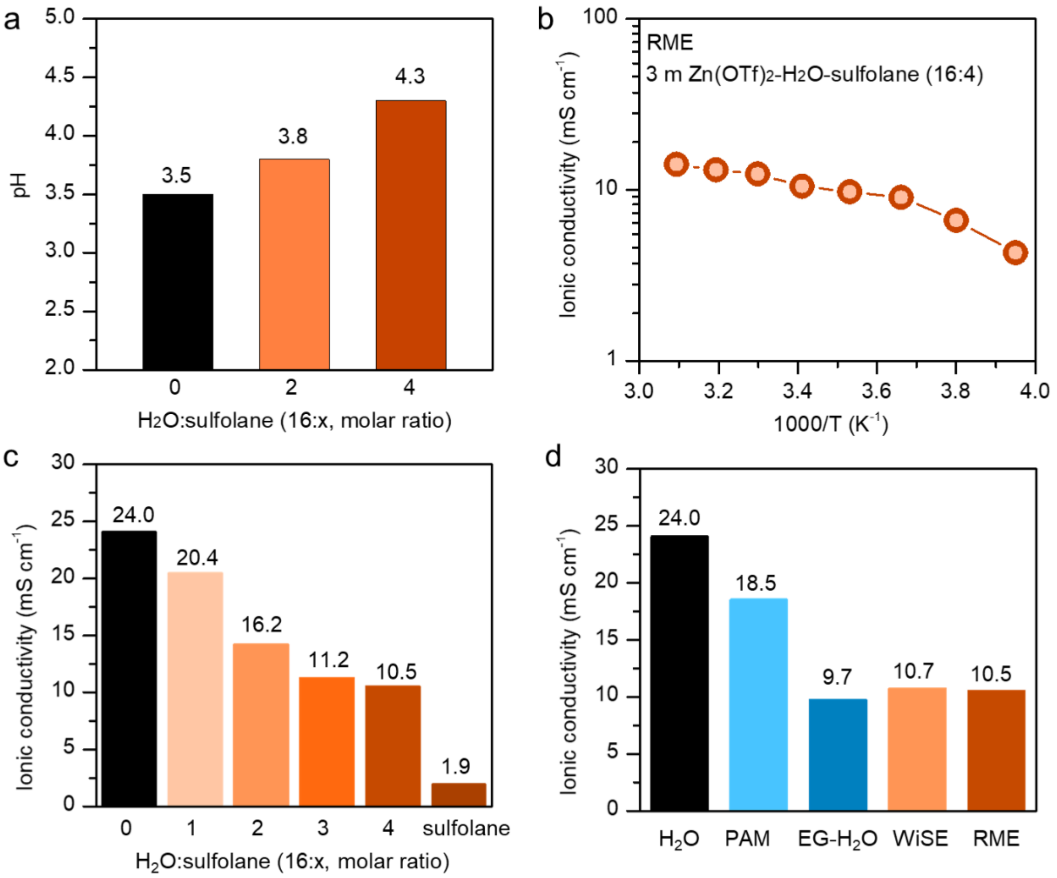

Supplementary Fig. 1 | The pH and ionic conductivity of the RME and various types of electrolytes. **a**, The pH of 3 m $Zn(OTf)_2$-H_2O-sulfolane (16:x, x=0, 2, 4, molar ratio) electrolytes. **b**, Arrhenius plot of the ionic conductivity for the RME at temperatures from −20 °C to 50 °C. **c**, The conductivity of the 3 m $Zn(OTf)_2$-H_2O-sulfolane (16:x, x=0, 1, 2, 3, 4) electrolytes and the 2 m $Zn(OTf)_2$/sulfolane electrolyte (denoted as sulfolane) at ~25°C. **d**, The conductivity of the H_2O, the PAM, the EG-H_2O, the WiSE, and the RME at ~25°C. Note that the solubility of $Zn(OTf)_2$ in the sulfolane solvent is lower than 3 m, we therefore show the conductivity of the 2 m $Zn(OTf)_2$/sulfolane electrolyte here.

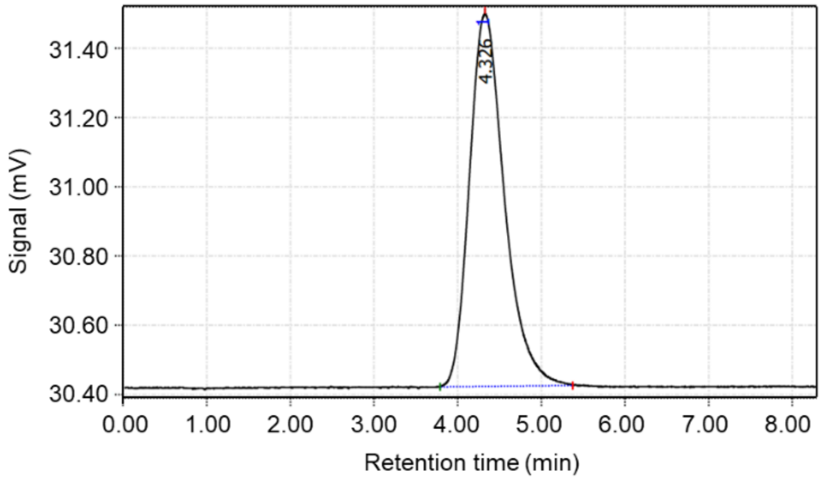

Supplementary Fig. 2 | Peak of H_2 gas detected by in situ GC during the LSV test in 3 m $Zn(OTf)_2/H_2O$ (H_2O) electrolyte. Here, the 3 m $Zn(OTf)_2/H_2O$ was selected to do the in situ GC test to qualitatively identify the major gas evolved as H_2.

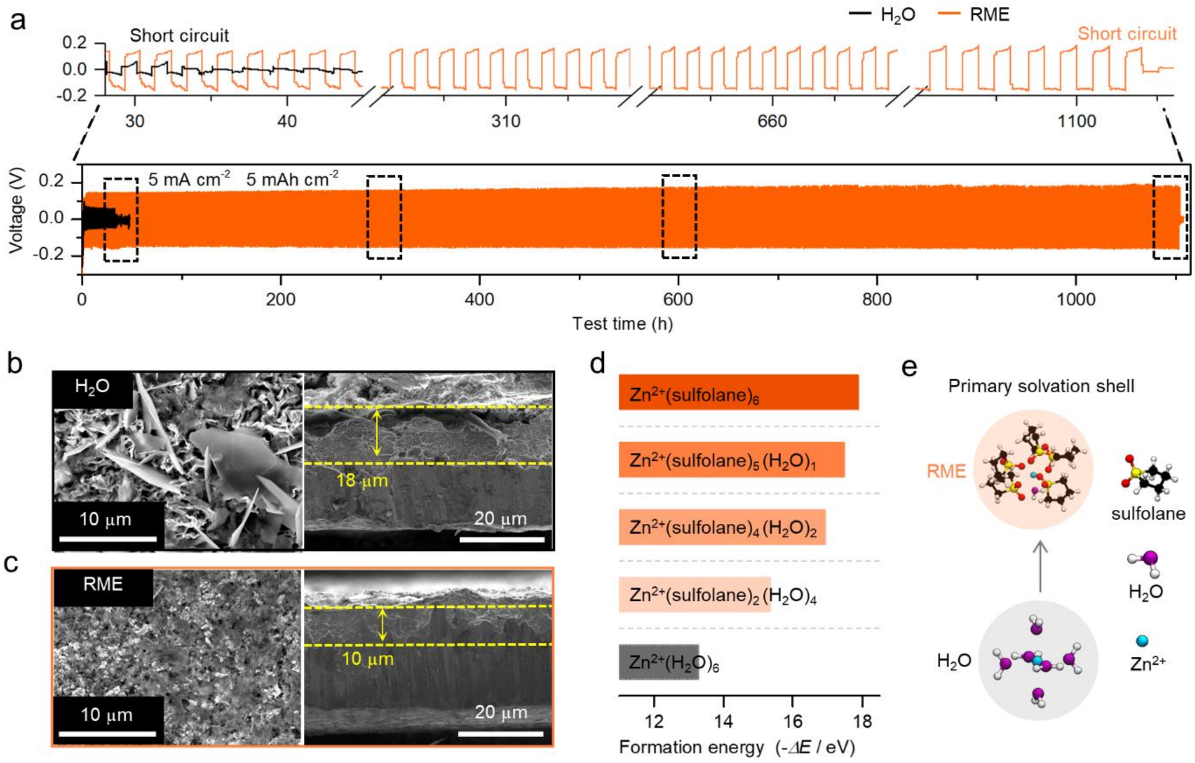

Supplementary Fig. 3 | Zn deposition stability in the H_2O electrolyte and the RME. a, Electrochemical performance of Zn||Zn symmetric cell in the 3 m $Zn(OTf)_2/H_2O$ (H_2O) electrolyte and the RME. The galvanostatic charging and discharging was conducted in the self-design electrode free standing cell to minimize the Zn loss on the current collector (see Supplementary Fig. 6 for the structure of the cell). The Zn foil with thickness of 30 µm was used here. **b**, SEM images of the deposited Zn^0 surface obtained in the H_2O electrolyte at 1 mA cm^{-2} for 5 mAh cm^{-2}. **c**, SEM images of deposited Zn^0 surface obtained in the RME at 1 mA cm^{-2} for 5 mAh cm^{-2}. **d**, Formation energy for various $Zn^{2+}(sulfolane)_n(H_2O)_{6-n}$ (n=6, 5, 4, 2, 0) complex. **e**, Primary solvation shell of the Zn^{2+} in the H_2O electrolyte and the RME. The tests were conducted at ~25 ℃.

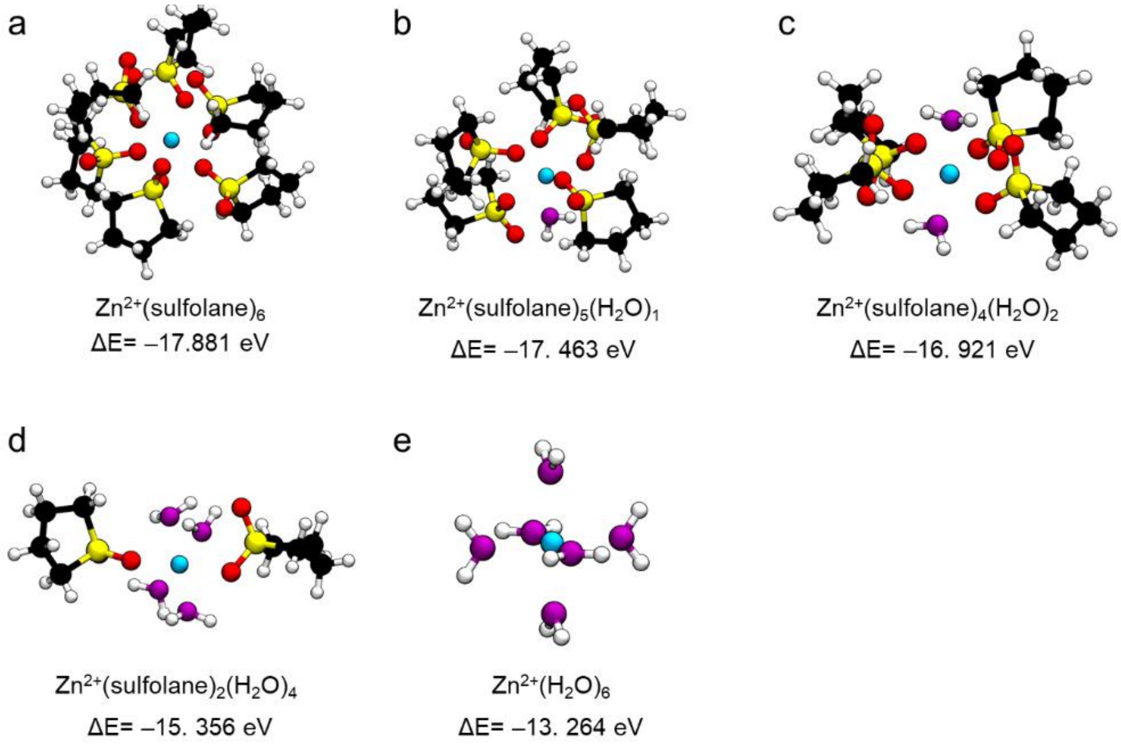

Supplementary Fig. 4 | DFT results of the primary solvation shell and corresponding formation energy of the Zn^{2+}(sulfolane)$_n$(H_2O)$_{6-n}$ (n=6, 5, 4, 2, and 0) complex. a-e, The primary solvation shell and corresponding formation energy of the Zn^{2+}(sulfolane)$_n$(H_2O)$_{6-n}$ complex where n=6 (**a**), 5 (**b**), 4 (**c**), 2 (**d**), and 0 (**e**). The black sphere represents carbon atom, the white sphere represents hydrogen atom, the yellow sphere represents sulfur atom, the red sphere represents the oxygen atom in sulfolane molecule, the light blue sphere represents the zinc ion, the purple represents the atom in water molecule.

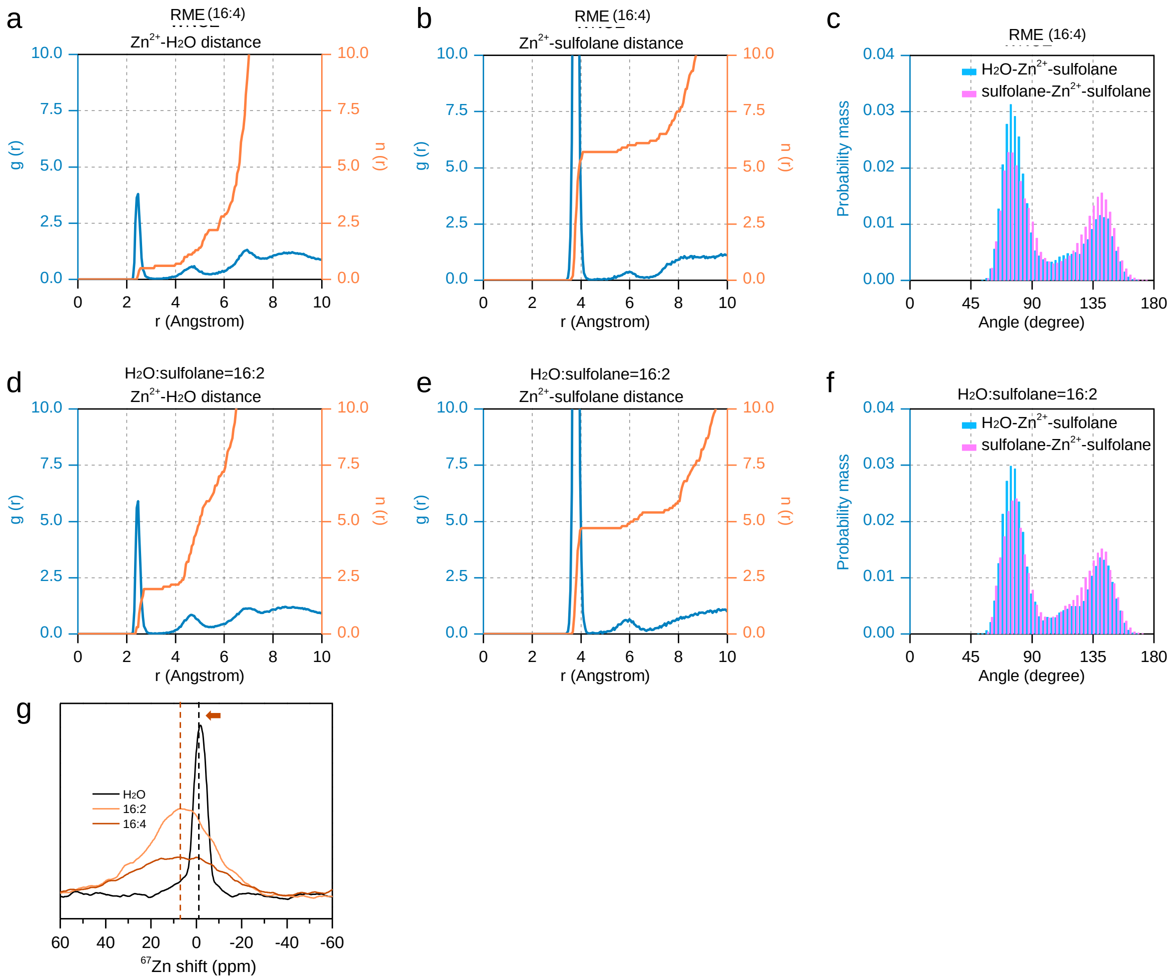

Supplementary Fig. 5 | MD simulations of the RME (16:4) and 3 m Zn(OTf)$_2$-H_2O-sulfolane (H_2O:sulfolane=16:2) electrolyte and the ^{16}Zn NMR spectra. a-b, The Zn^{2+}-H_2O and Zn^{2+}-sulfolane radial distribution function (g(r)) and coordination numbers (n(r)). **c**, The probability of the angle for H_2O-Zn^{2+}-sulfolane and sulfolane-Zn^{2+}-sulfolane. **d-e**, The Zn^{2+}-H_2O and Zn^{2+}-sulfolane radial distribution function (g(r)) and coordination numbers (n(r)) in the 3 m Zn(OTf)$_2$-H_2O-sulfolane (H_2O:sulfolane=16:2) electrolyte. **f**, The probability of the angle for H_2O-Zn^{2+}-sulfolane and sulfolane-Zn^{2+}-sulfolane in the 3 m Zn(OTf)$_2$-H_2O-sulfolane (H_2O:sulfolane=16:2) electrolyte. **g**, The ^{67}Zn NMR spectra.

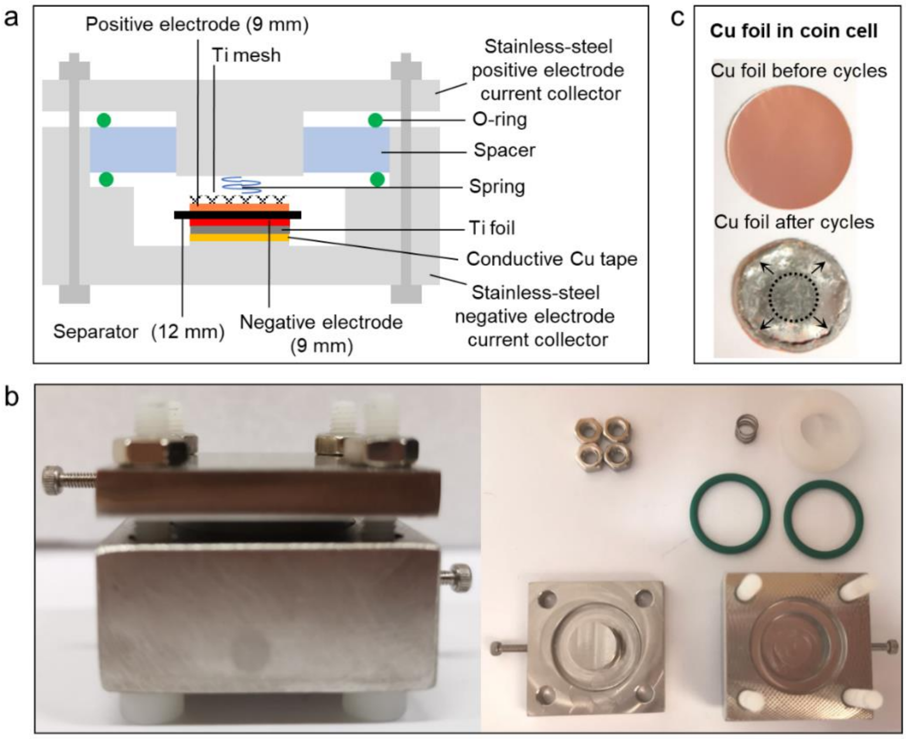

Supplementary Fig. 6 | The configuration of the in-house developed electrode free standing cell . a, Structure of the electrode free standing cell. **b**, The photos of the free standing cell. Here the right part is the cell before assembly and the left part is the cell after assembly. **c**, Comparison of Cu foil before and after cycling in coin cell. The free standing cell enables the electrode area identical to current collector avoiding the Zn spreading on current collector (Supplementary Fig. 6c) upon deposition thus minimizing the Zn loss on current collector. The contact area between stainless-steel negative current collector and Zn foil was wrapped by a piece of conductive Cu tape to minimize the side reactions between electrolyte and stainless-steel current collector. A piece of Ti foil was placed between the copper tape and Zn foil to minimize the side reactions between Zn foil and Cu tape.

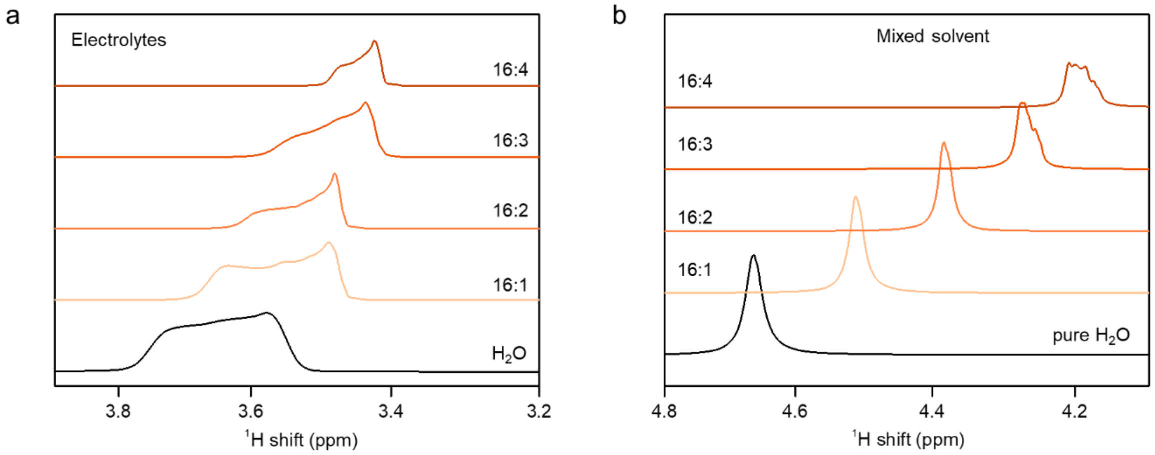

Supplementary Fig. 7 | The ^{1}H NMR spectra for various electrolytes and solvent. a, The ^{1}H NMR spectra of the 3 m $Zn(OTf)_2/H_2O$ (H_2O) and 3 m $Zn(OTf)_2$-H_2O-sulfolane (H_2O:sulfolane=16:x, x=1, 2, 3, 4) electrolytes. **b**, The ^{1}H NMR spectra for the H_2O:sulfolane (16:x, x=1, 2, 3, 4) mixed solvents. Note that the broadening of the peaks in Supplementary Fig. 7a was caused by the influence of $Zn(OTf)_2$ salt in the electrolytes. The ^{1}H NMR spectra for the pure H_2O and mixed solvents without salt were collected to further validate the formation of hydrogen bond between sulfolane and water.

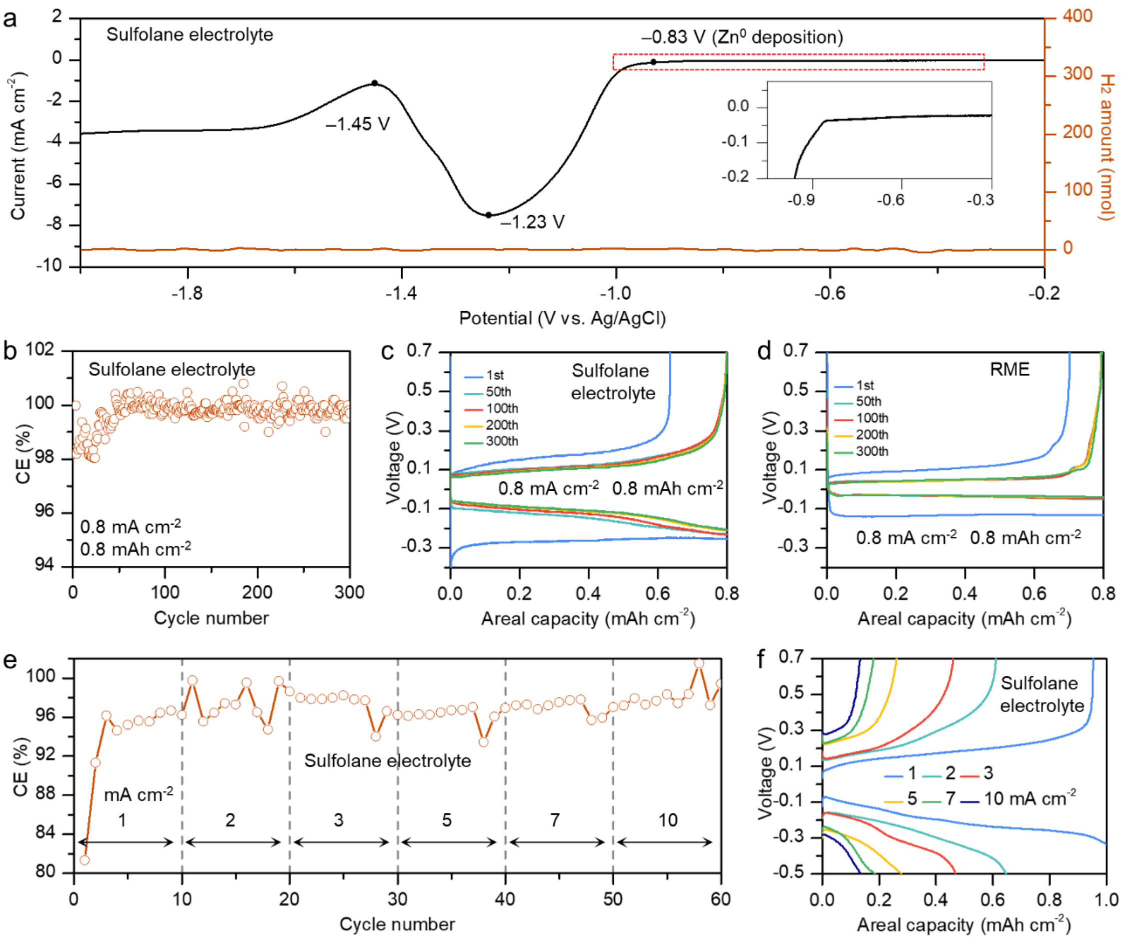

Supplementary Fig. 8 | The Zn^0 plating performance in the 2 m $Zn(OTf)_2$/sulfolane electrolyte (denoted as sulfolane electrolyte). a, Cathodic stability and H_2 coevolution behavior of the sulfolane electrolyte determined by LSV and operando gas pressure measurement. **b**, The CE of Zn||Cu asymmetric cell with the sulfolane electrolyte. **c**, The galvanostatic charging and discharging profiles of the Zn||Cu asymmetric cell in the sulfolane electrolyte. **d**, The galvanostatic charging and discharging profiles of the Zn(50 μm)||Cu asymmetric cell in the RME. **e**, The CE of the Zn||Cu asymmetric cell in the sulfolane electrolyte at different current densities. **f**, The galvanostatic charging and discharging profiles of the Zn||Cu asymmetric cell in the sulfolane electrolyte at different current densities. The Zn foil with thickness of 50 μm was used here. The tests were conducted at ~25 ℃.

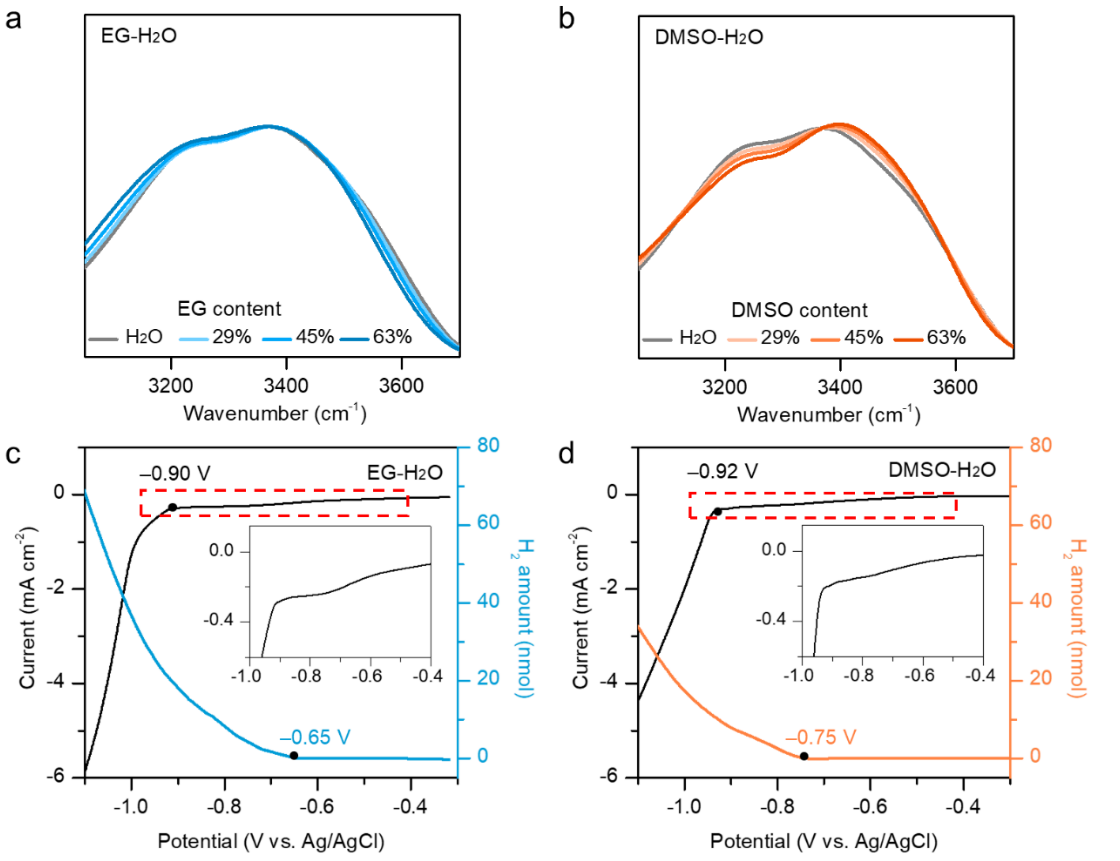

Supplemental Fig. 9 | The FTIR spectra and the cathodic stability of the EG-H_2O and the DMSO-H_2O electrolytes. a, Normalized FTIR spectra of the H_2O electrolyte and 3 m $Zn(OTF)_2$-(1-x)H_2O-xEG (x=29%, 45%, 63%, weight content) electrolytes. **b**, Normalized FTIR spectra of the H_2O electrolyte and 3 m $Zn(OTF)_2$-(1-x)H_2O-xDMSO (x=29%, 45%, 63%, weight content) electrolytes. **c**, Cathodic stability and H_2 coevolution behavior for the 3 m $Zn(OTF)_2$-37%H_2O-63%EG (denoted as EG-H_2O) electrolyte determined by LSV and operando gas pressure measurement. **d**, Cathodic stability and H_2 coevolution behavior for the 3 m $Zn(OTF)_2$-37%H_2O-63%DMSO (denoted as DMSO-H_2O) electrolyte determined by LSV and operando gas pressure measurement. For a fair comparison, the EG and DMSO were set as the same weight content with the sulfolane where the 3 m $Zn(OTf)_2$-H_2O-sulfolane (16:x, x=1, 2, 4) electrolytes correspond to 3 m $Zn(OTF)_2$-(1-x)H_2O-x (sulfolane) (x=29%, 45%, 63%, weight content). The tests were conducted in a three-electrode cell consisting of Ti foil working electrode, AC counter electrode, and Ag/AgCl reference electrode at ~25 °C.

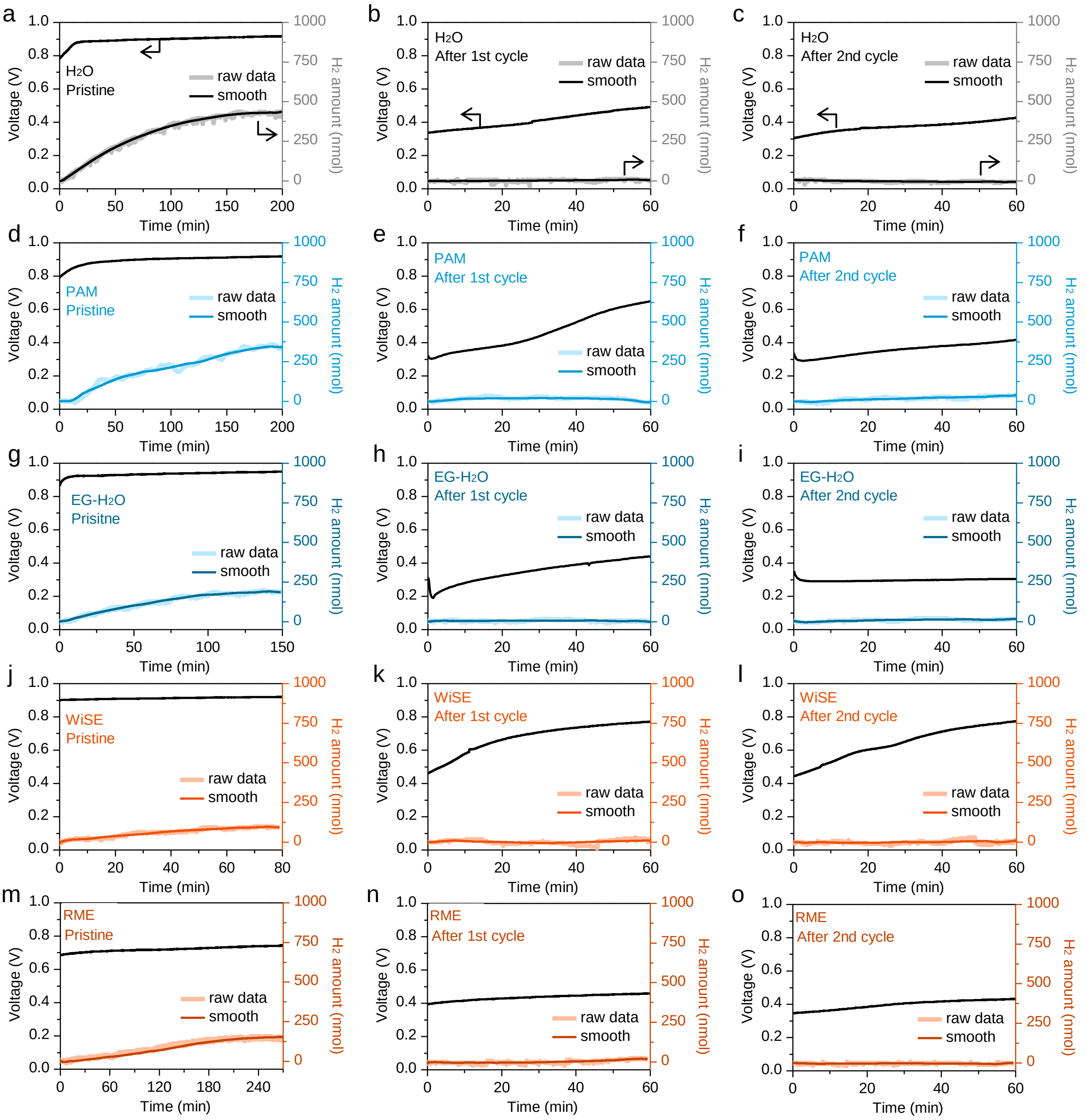

Supplementary Fig. 10 | H_2 gas evolution from corrosion effect between Zn^0 anode and various electrolytes. a-c, H_2 gas evolution between Zn^0 anode and the H_2O electrolyte at pristine state, after 1st cycle, and after 2nd cycle. **d-f**, H_2 gas evolution between Zn^0 anode and the PAM at pristine state, after 1st cycle, and after 2nd cycle. **g-i**, H_2 gas evolution between Zn^0 anode and the EG-H_2O electrolyte at pristine state, after 1st cycle, and after 2nd cycle. **j-l**, H_2 gas evolution between Zn^0 anode and the WiSE at pristine state, after 1st cycle, and after 2nd cycle. **m-o**, H_2 gas evolution between Zn^0 anode and the RME at pristine state, after 1st cycle, and after 2nd cycle. The H_2 gas evolutions at the pristine state were recorded by holding the cell at OCV until the gas pressure reaching to a relative stable state (200 min for the H_2O and the PAM electrolyte, 150 min for the EG-H_2O electrolyte, 80 min for the WiSE, and 270 min for the RME). The H_2 gas evolutions after the first and second cycle were recorded by holding the cell at OCV for 60 min. The tests were conducted in a three-electrode cell consisting of Ti foil working electrode, AC counter electrode, and Ag/AgCl reference electrode at ~25 °C.

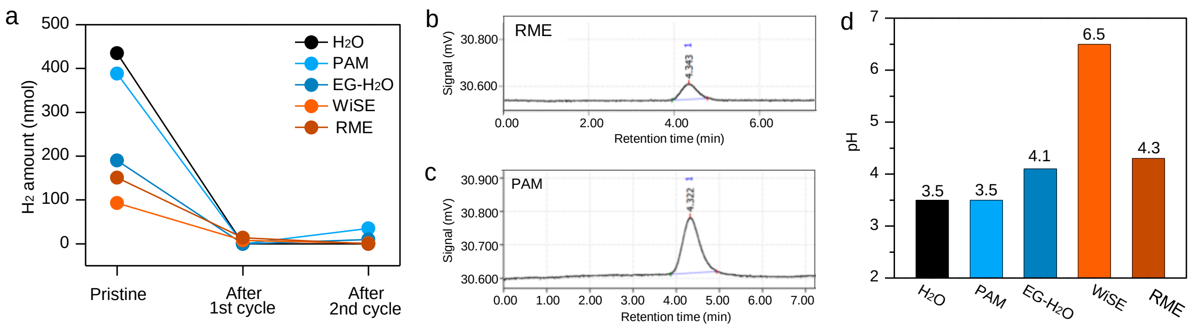

Supplementary Fig. 11 | H_2 gas evolution from corrosion effect between Zn^0 anode and various electrolytes. a, H_2 gas evolution between Zn^0 anode and the H_2O, the PAM, the EG-H_2O, the WiSE, and the RME in pristine state, after the first cycle and after the second cycle. **b**, Peak of H_2 gas detected by in situ GC in the RME. **c**, Peak of H_2 gas detected by in situ GC in the PAM. Here, the RME and the PAM were selected to do the in situ GC measurement to qualitatively identify the major gas evolved as H_2. **d**, The pH values of the H_2O, the PAM, the EG-H_2O, the WiSE, and the RME. The tests were conducted in a three-electrode cell consisting of Ti foil working electrode, AC counter electrode, and Ag/AgCl reference electrode at ~25 °C.

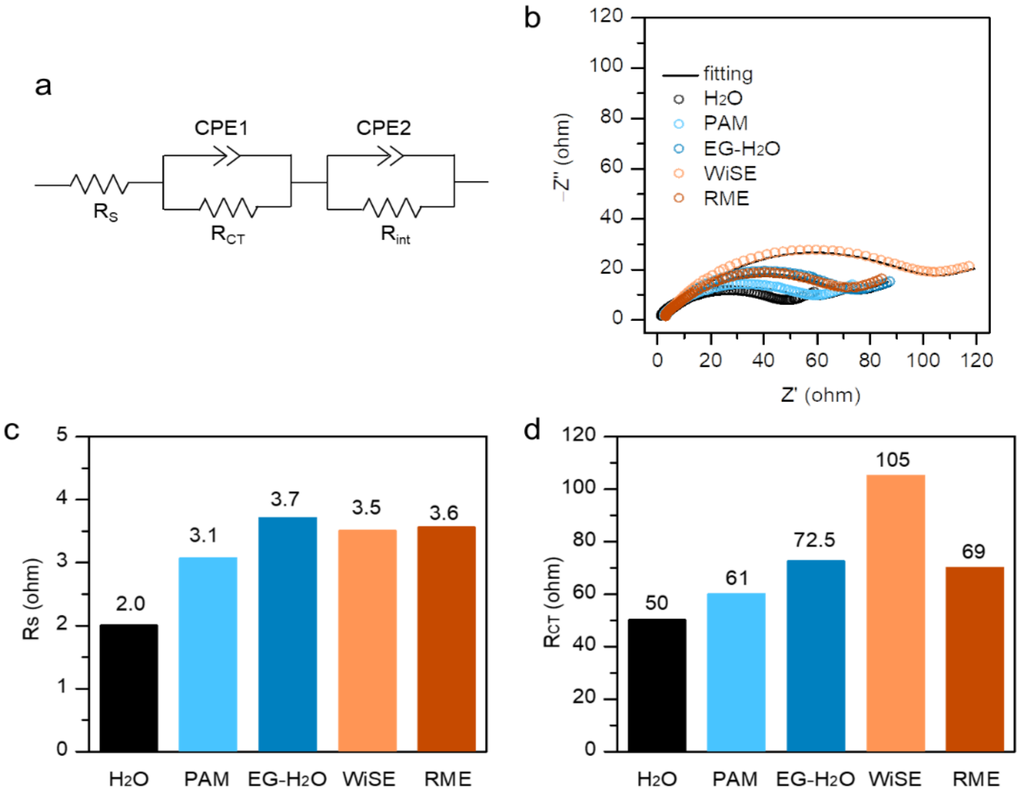

Supplementary Fig. 12 | The impedance of the Zn||Zn cell in various electrolytes. a, The equivalent circuit model for data fitting. **b**, The original EIS data and fitting curves. **c**, The ohmic resistance. **d**, The charge transfer resistance. The EIS data were collected after ten galvanostatic cycles at 0.8 mA cm^{-2} and 0.8 mAh cm^{-2} where the system has reached a relatively stable state. The fitting model was referenced from a previous study[1]. The tests were conducted at ~25 °C.

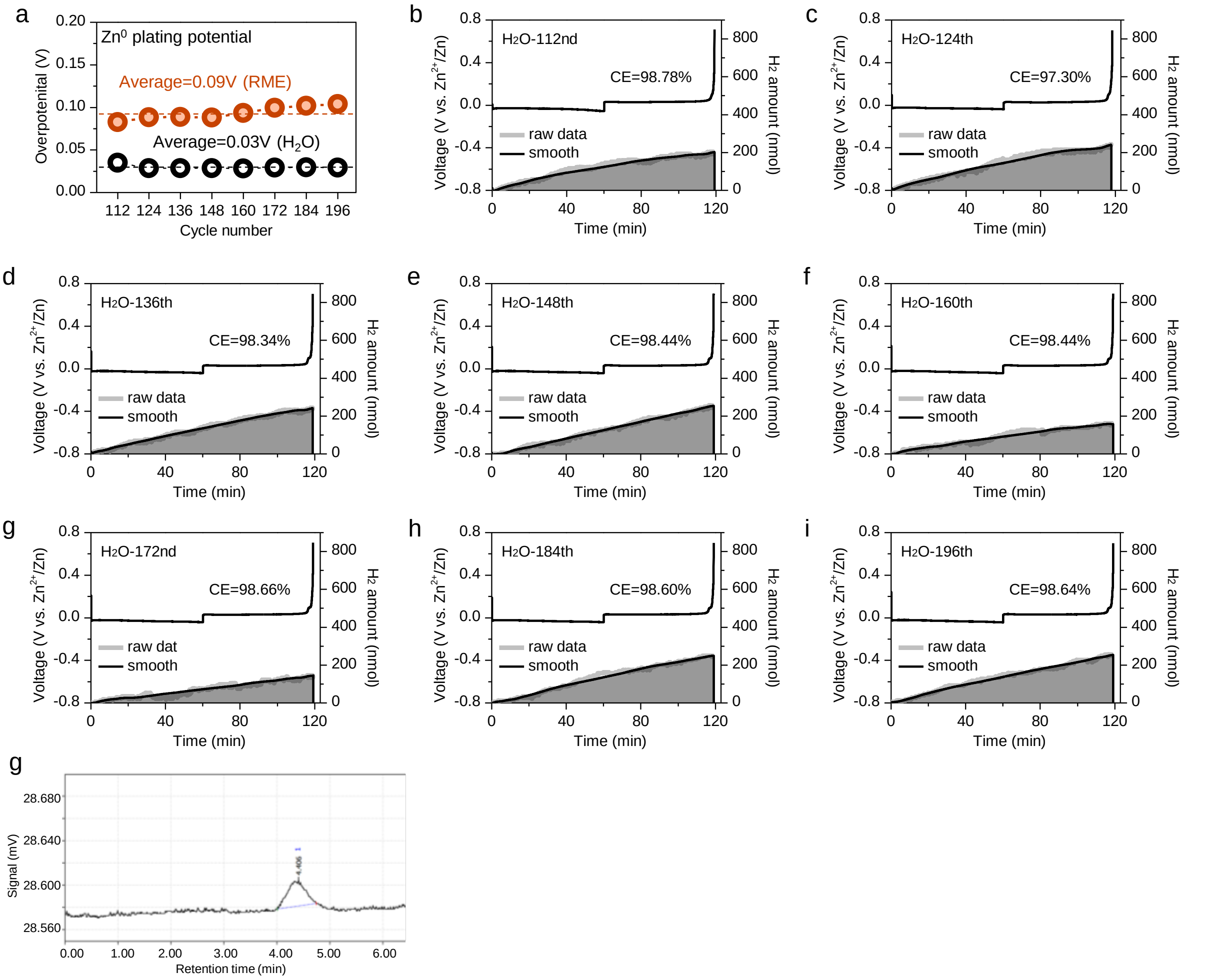

Supplementary Fig. 13 | Galvanostatic cycles and operando gas evolution in the H_2O electrolyte. a, The comparison of the Zn^0 plating potential in the RME and the H_2O electrolyte. **b-g**, Galvanostatic discharging and charging profiles and H_2 evolution for the 112nd (**b**), 124th (**c**), 136th (**d**), 148th (**e**), 160th (**f**), 172nd (**g**), 184th (**h**), and 196th (**i**) cycle. **g**, Peak of H_2 gas detected by in situ GC during the galvanostatic discharging in the H_2O electrolyte in 112nd cycle. Here, the H_2O electrolyte was selected to do the in situ GC measurement to qualitatively identify the major gas evolved as H_2. The galvanostatic charging and discharging were conducted at constant current of 0.8 mA cm^{-2} with capacity of 0.8 mAh cm^{-2}. The tests were conducted in the three-electrode cell consisting of Ti foil working electrode, AC counter electrode, and Ag/AgCl reference electrode at ~25 ℃.

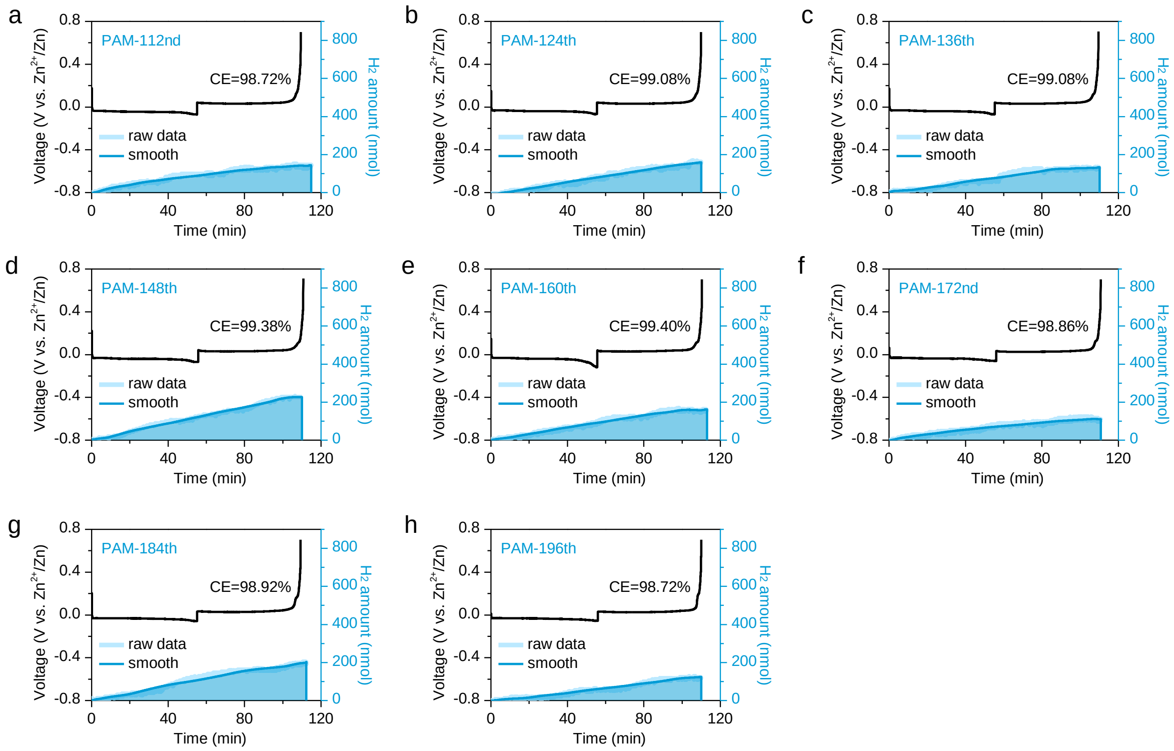

Supplementary Fig. 14 | Galvanostatic cycles and operando gas evolution in the PAM electrolyte. a-h, Galvanostatic discharging and charging profiles and H_2 evolution for the 112nd (**a**), 124th (**b**), 136th (**c**), 148th (**d**), 160th (**e**), 172nd (**f**), 184th (**g**), and 196th (**h**) cycle. The galvanostatic charging and discharging were conducted at constant current of 0.8 mA cm^{-2} with capacity of 0.8 mAh cm^{-2}. The tests were conducted in a three-electrode cell consisting of Ti foil working electrode, AC counter electrode, and Ag/AgCl reference electrode at ~25 °C.

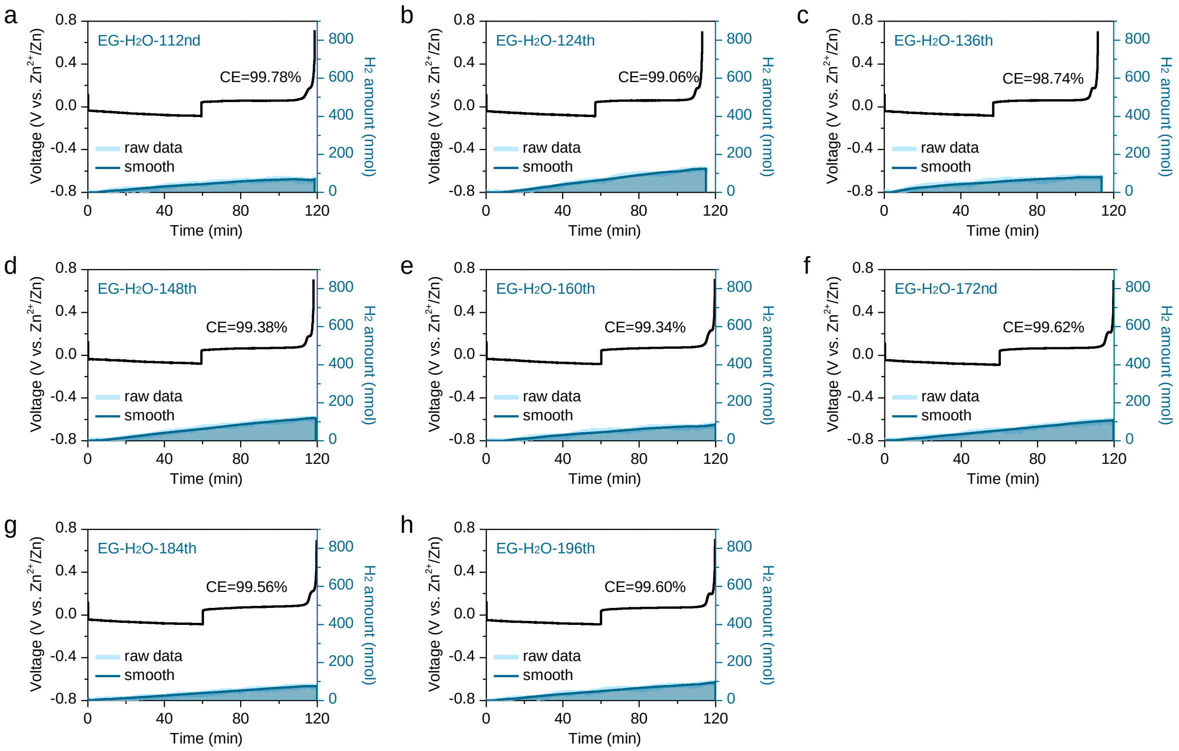

Supplementary Fig. 15 | Galvanostatic cycles and operando gas evolution in the EG-H_2O electrolyte. a-h, Galvanostatic discharging and charging profiles and H_2 evolution for the 112nd (**a**), 124th (**b**), 136th (**c**), 148th (**d**), 160th (**e**), 172nd (**f**), 184th (**g**), and 196th (**h**) cycle. The galvanostatic charging and discharging were conducted at constant current of 0.8 mA cm^{-2} with capacity of 0.8 mAh cm^{-2}. The tests were conducted in a three-electrode cell consisting of Ti foil working electrode, AC counter electrode, and Ag/AgCl reference electrode at ~25 °C.

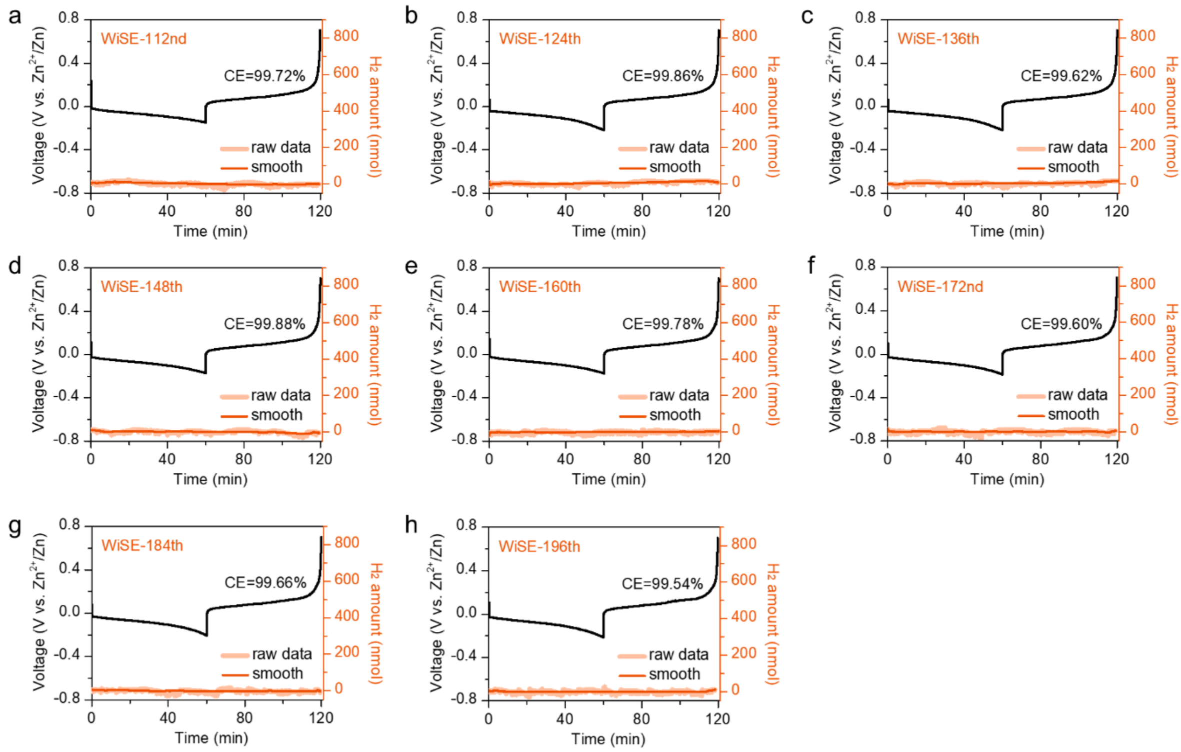

Supplementary Fig. 16 | Galvanostatic cycles and operando gas evolution in the WiSE. a-h, Galvanostatic discharging and charging profiles and H_2 evolution for the 112nd (**a**), 124th (**b**), 136th (**c**), 148th (**d**), 160th (**e**), 172nd (**f**), 184th (**g**), and 196th (**h**) cycle. The galvanostatic charging and discharging were conducted at constant current of 0.8 mA cm^{-2} with capacity of 0.8 mAh cm^{-2}. The tests were conducted in a three-electrode cell consisting of Ti foil working electrode, AC counter electrode, and Ag/AgCl reference electrode at ~25 °C.

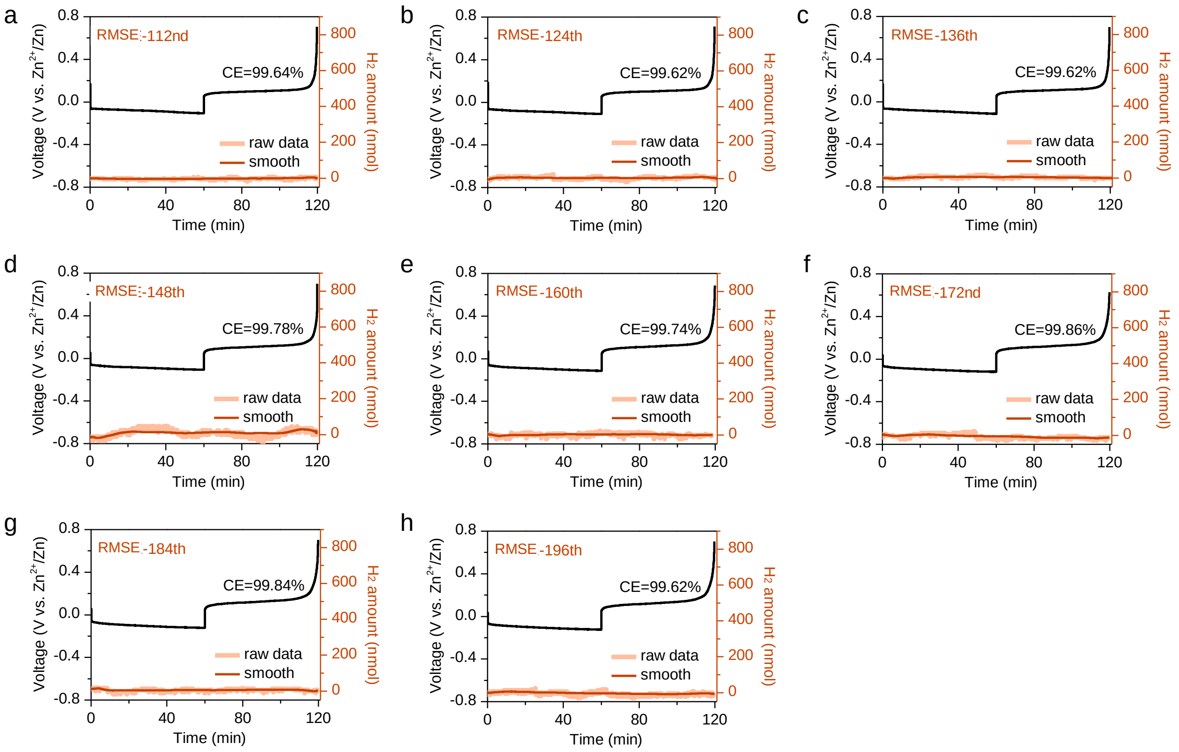

Supplementary Fig. 17 | Galvanostatic cycles and operando gas evolution in the RME. a-h, Galvanostatic discharging and charging profiles and H_2 evolution for the 112nd (**a**), 124th (**b**), 136th (**c**), 148th (**d**), 160th (**e**), 172nd (**f**), 184th (**g**), and 196th (**h**) cycle. The galvanostatic charging and discharging were conducted at constant current of 0.8 mA cm^{-2} with capacity of 0.8 mAh cm^{-2}. The tests were conducted in a three-electrode cell consisting of Ti foil working electrode, AC counter electrode, and Ag/AgCl reference electrode at ~25 °C.

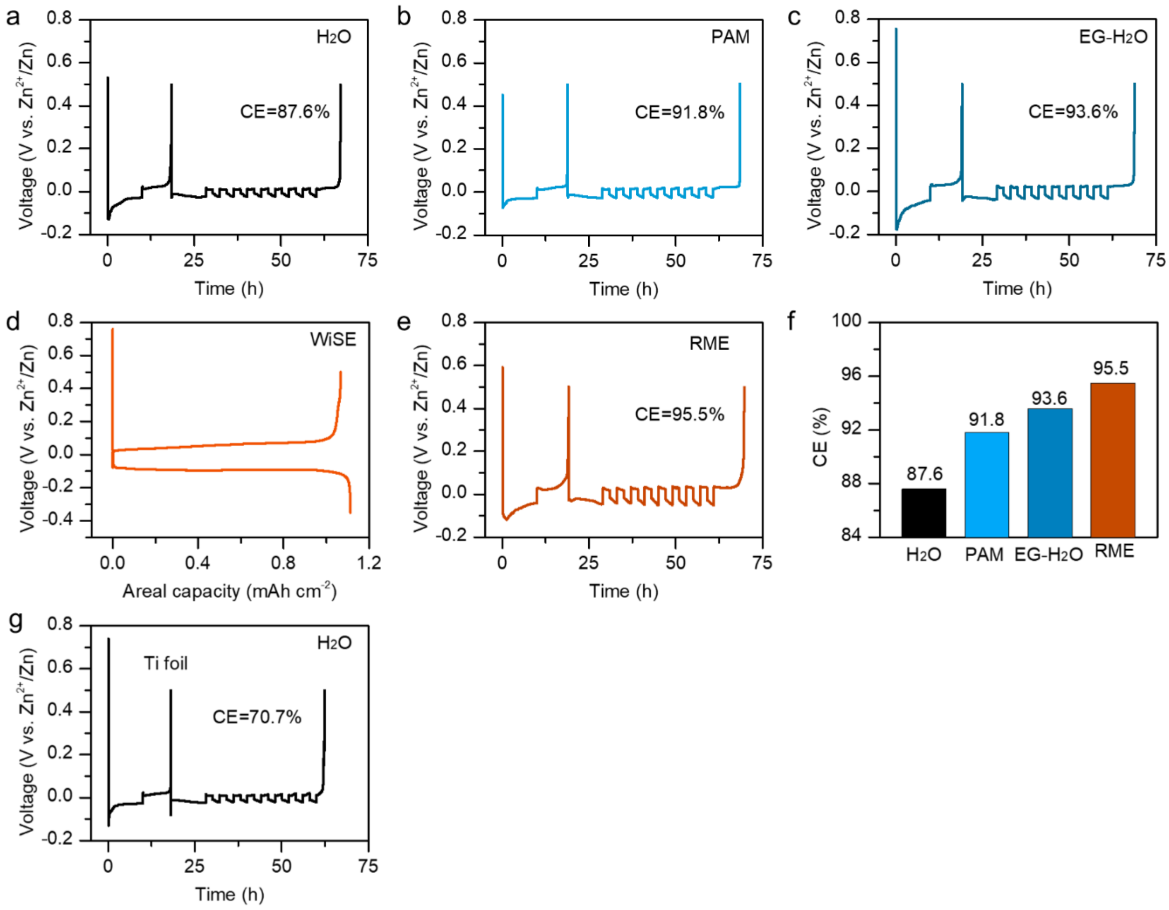

Supplementary Fig. 18 | CE of the Zn||Cu or the Zn||Ti cell in various electrolytes. a, CE of the Zn||Cu cell in the H_2O electrolyte. **b**, CE of the Zn||Cu cell in the PAM electrolyte. **c**, CE of the Zn||Cu cell in the EG-H_2O electrolyte. **d**, CE of the Zn||Cu cell in the WiSE. **e**, CE of the Zn||Cu cell in the RME. **f**, The comparison of CEs of the Zn||Cu cell in the five electrolytes. **g**, CE of the Zn||Ti cell in H_2O electrolyte. Note that the Cu electrode was firstly conditioned by plating (5 mAh cm^{-2}) and stripping Zn^0 (0.5 V). Then a Zn^0 reservoir with a capacity of 5 mAh cm^{-2} was plated on the Cu electrode at 0.5 mA cm^{-2}. Then the cell was stripping/plating for 8 cycles at 0.5 mA cm^{-2} with 1 mAh cm^{-2} . In the final step, all the capacity plated on Cu electrode was stripped with cut-off voltage of 0.5 V. The CEs labelled in each panel were calculated by the previously reported method[2]. The Zn foil used here is 100 µm. The WiSE cannot sustain the areal capacity of 5 mAh cm^{-2} as shown in Supplementary Fig. 18d. The Zn||Ti cell exhibits a lower CE than the Zn||Cu cell, as shown in Supplementary Fig. 18g and Supplementary Fig. 18a, therefore, Cu foil was used as metal substrate for the Zn^0 plating/stripping in this work. The tests were conducted at ~25 °C.

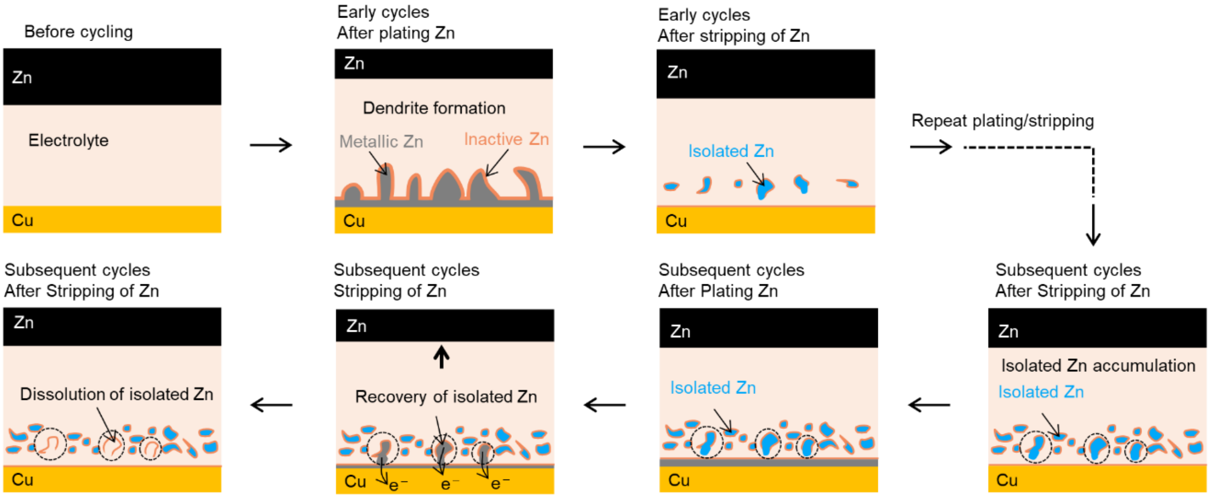

Supplementary Fig. 19 | Schematic illustration for the generation and recovery of the isolated Zn.

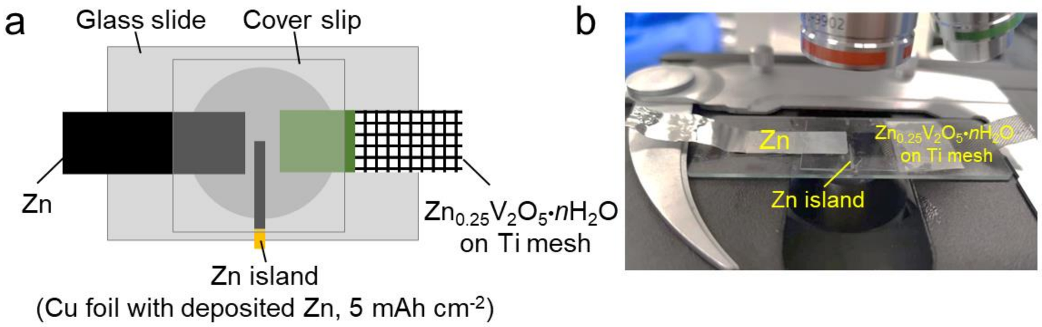

Supplementary Fig. 20 | Schematic illustration and the photo of the in situ optical microscopy cell. a, The schematic illustration of the in situ optical microscopy cell with Zn foil (50 µm) as anode, $Zn_{0.25}V_2O_5{\bullet}nH_2O$ as cathode, 3 m $Zn(OTf)_2/H_2O$ as electrolyte and a Cu foil deposited with Zn metal (5 mAh cm^{-2}) as Zn-island (width, ~200 µm). **b**, The photo of the in situ optical microscopy cell. The test was conducted at ~25 °C.

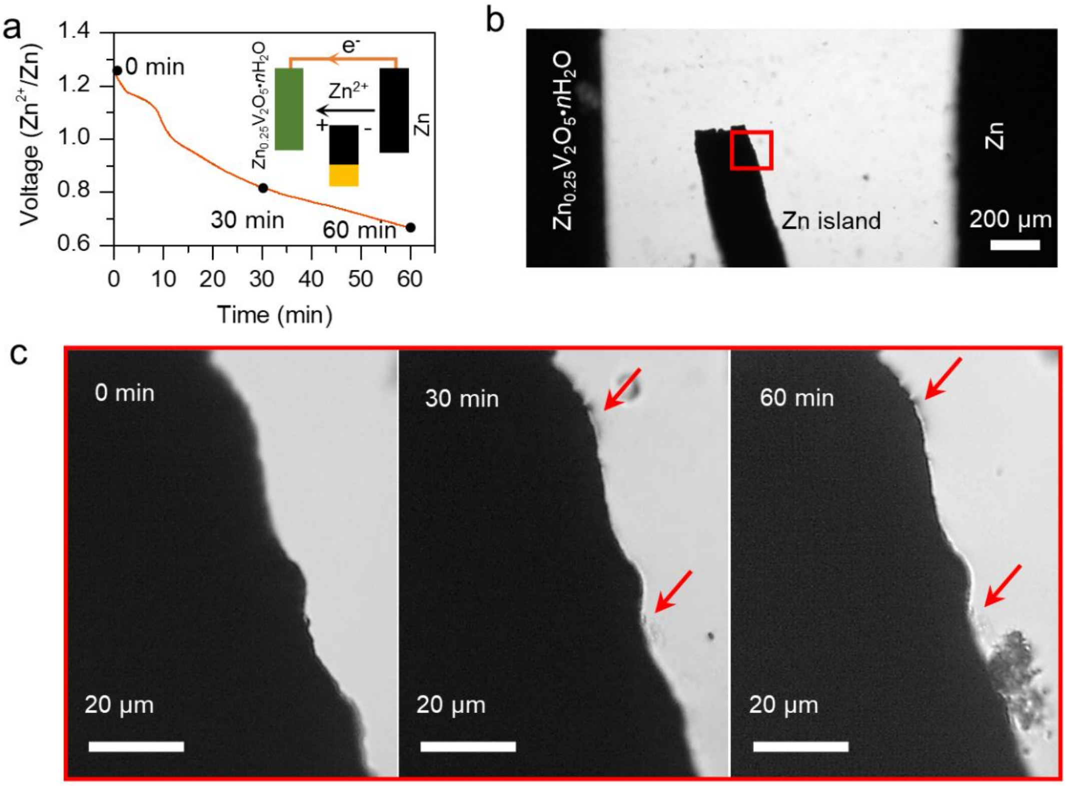

Supplementary Fig. 21 | Morphology evolution of the isolated Zn island. a, The galvanostatic discharging profile of the in situ optical microscopy cell. The current is 300 µA. **b**, The optical image of the cell under the 5 × lens. **c**, The optical images of the Zn-island (close to Zn foil) at different discharging time under the 50 × lens. The test was conducted at ~25 ℃ with the cell configuration in Supplementary Fig. 20a.

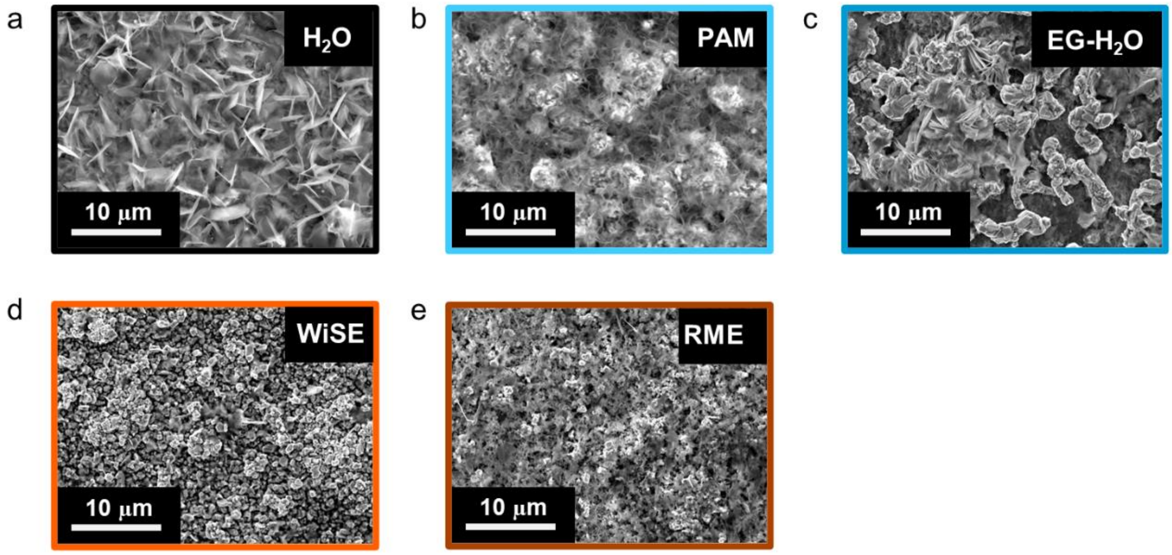

Supplementary Fig. 22 | Ex situ SEM images for the deposited Zn^0 surface in various electrolytes. **a**, SEM image for the deposited Zn^0 surface in the H_2O electrolyte. **b**, SEM image for the deposited Zn^0 surface in the PAM electrolyte. **c**, SEM image for the deposited Zn^0 surface in the EG-H_2O electrolyte. **d**, SEM image for the deposited Zn^0 surface in the WiSE. **e**, SEM image for the deposited Zn^0 surface in the RME. The SEM samples were obtained by discharging and charging in Zn||Zn cell for 20 cycles at 0.8 mA cm^{-2} with 0.8 mAh cm^{-2} and finally depositing Zn^0 with 1 mAh cm^{-2}. The free standing cell was used here to prepared the ex situ SEM samples. The cells were tested at ~25 ℃.

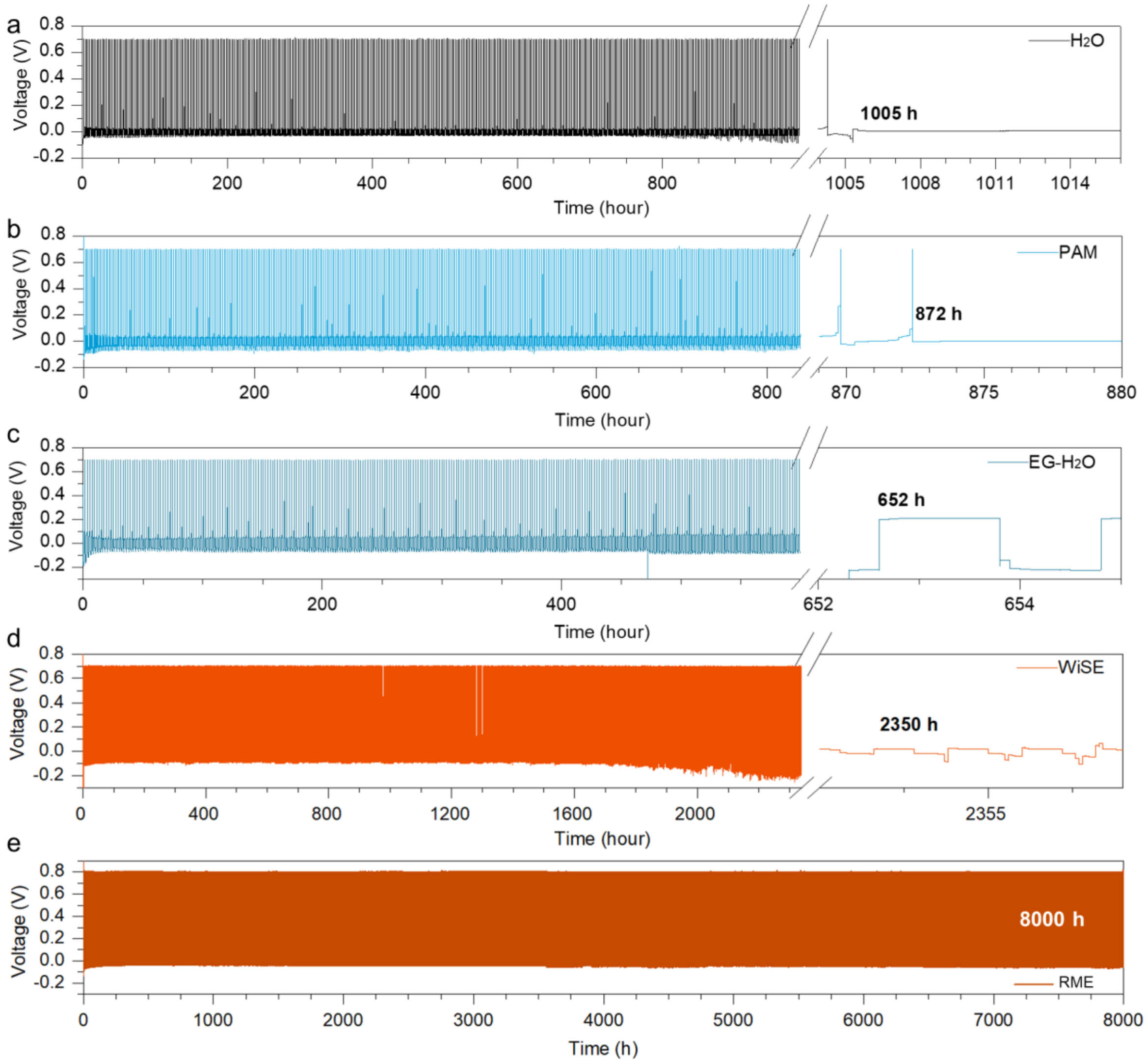

Supplementary Fig. 23 | Voltage profiles for the Zn||Cu cell in various electrolytes. a, Voltage curve as a function of time for the Zn||Cu cell in the H_2O electrolyte. **b**, Voltage curve as a function of time for the Zn||Cu cell in the PAM. **c**, Voltage curve as a function of time for the Zn||Cu cell in the EG-H_2O electrolyte. **d**, Voltage curve as a function of time for the Zn||Cu cell in the WiSE. **e**, Voltage curve as a function of time for the Zn||Cu cell in the RME. The tests were performed with the electrode free standing cell (Supplementary Fig. 6). The Zn foil with thickness of 50 μm was used here. The galvanostatic charging and discharging were conducted at constant current of 0.8 mA cm^{-2} with capacity of 0.8 mAh cm^{-2}. The tests were conducted at ~25 °C.

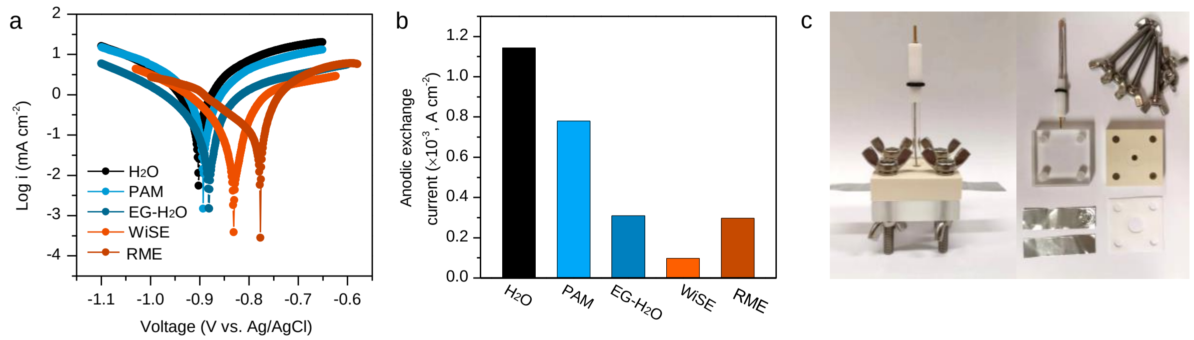

Supplementary Fig. 24 | Tafel behaviors in various electrolytes. a, Tafel plots in the H_2O, the PAM, the EG-H_2O, the WiSE, and the RME. **b**, The comparison for anodic exchange current in the H_2O, the PAM, the EG-H_2O, the WiSE, and the RME. **c**, The photos of the three electrode cell used to collect the Tafel plots. The Tafel plot was obtained by using Zn foil (50 μm) as working and counter electrode, Ag/AgCl as reference electrode at scan rate of 1 mV s^{-1}. The anodic exchange current density was used to assess the reaction kinetics as less parasitic H_2 coevolution would occur in anodic processes. The tests were conducted at ~25 °C.

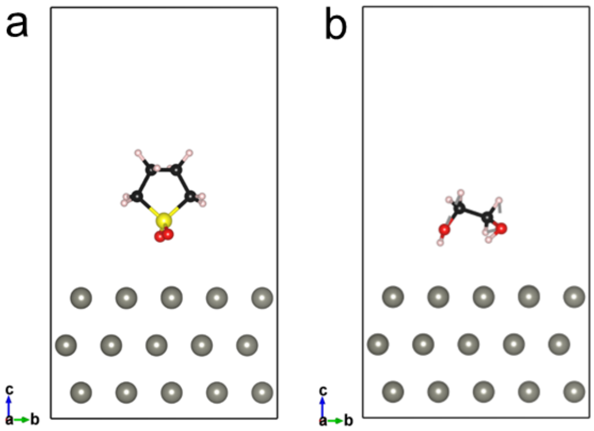

Supplementary Fig. 25 | The adsorption behaviors of the sulfolane and EG molecule on Zn surface. a, The adsorption behavior of the sulfolane molecule on Zn surface. **b**, The adsorption behavior of the EG molecule on Zn surface. The adsorption energy of EG (−1.28 eV) is closed to that of the sulfolane (−1.31 eV), however, the side reactions in the EG-H_2O electrolyte are more serious than that in the RME. This suggests the dominant role of "reverse micelle" structure in improving the reversibility of the Zn^0 electrode instead of molecule adsorption. The grey sphere represents the zinc atom, the red sphere represents the oxygen atom, the yellow sphere represents the sulfur atom, the black sphere represents the carbon atom, and the pink sphere represents the hydrogen atom.

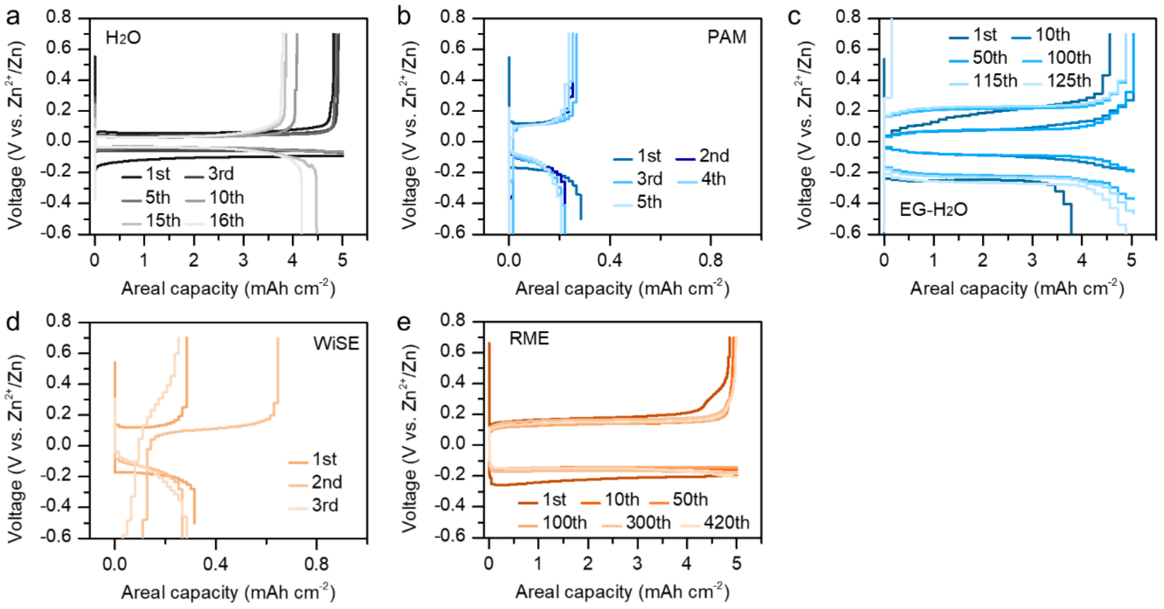

Supplementary Fig. 26 | Galvanostatic discharging and charging profiles of the Zn||Cu cell in various electrolytes. a, Galvanostatic discharging and charging profiles of the Zn||Cu cell in the H_2O electrolyte (**a**), the PAM electrolyte (**b**), the EG-H_2O electrolyte (**c**), the WiSE (**d**), and the RME (**e**). The tests were conducted in the electrode free standing cell (Supplementary Fig. 6) at 5 mA cm^{-2} and 5 mAh cm^{-2} with Zn utilization ratio of 28.3% (Zn foil, 30 µm). The tests were conducted at ~25 °C.

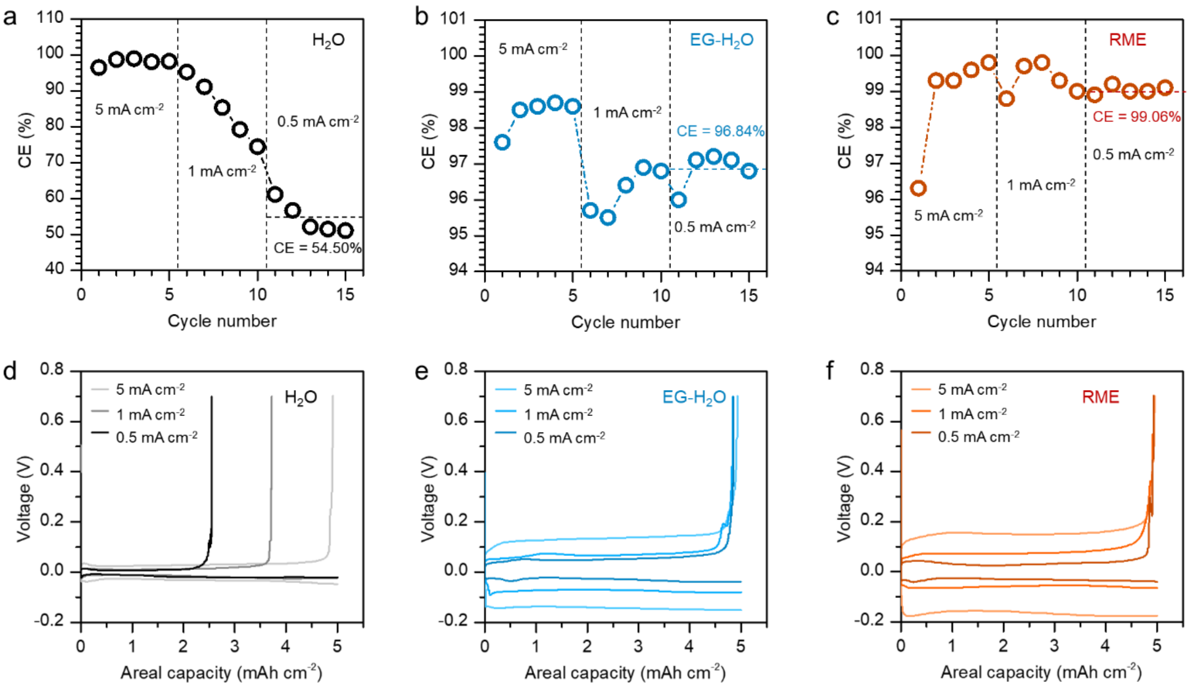

Supplementary Fig. 27 | The Zn||Cu cell performance at various rates. The CEs of the Zn||Cu cell in the H_2O (**a**), the EG-H_2O (**b**), and the RME (**c**). The galvanostatic discharging and charging profiles of the Zn||Cu cell in the H_2O (**d**), the EG-H_2O (**e**), and the RME (**f**). The tests were conducted in the electrode free standing cell (Supplementary Fig. 6) at 5 mAh cm^{-2} with Zn utilization ratio of 28.3% (Zn foil, 30 μm). The tests were conducted at ~25 °C.

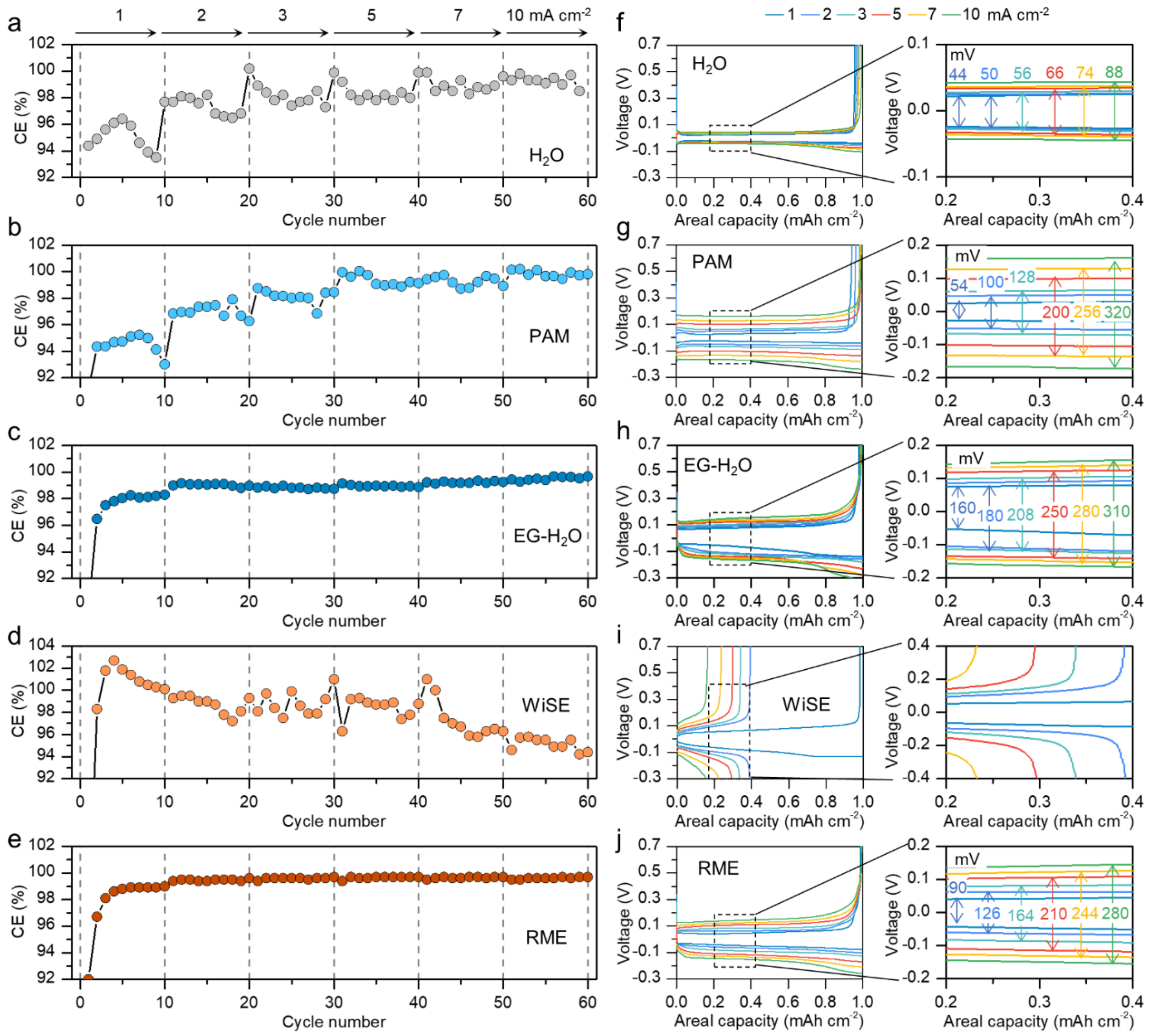

Supplemental Fig. 28 | The high-rate performance of Zn||Cu cell in various types of electrolytes. a−e, The CE under different current densities in the H_2O (**a**), the PAM (**b**), the EG-H_2O (**c**), the WiSE (**d**), and the RME (**e**). **f−j**, The galvanostatic charging and discharging profiles of the Zn||Cu cell in the H_2O (**f**), the PAM (**g**), the EG-H_2O (**h**), the WiSE (**i**), and the RME (**j**). The tests were conducted at ~25 ℃.

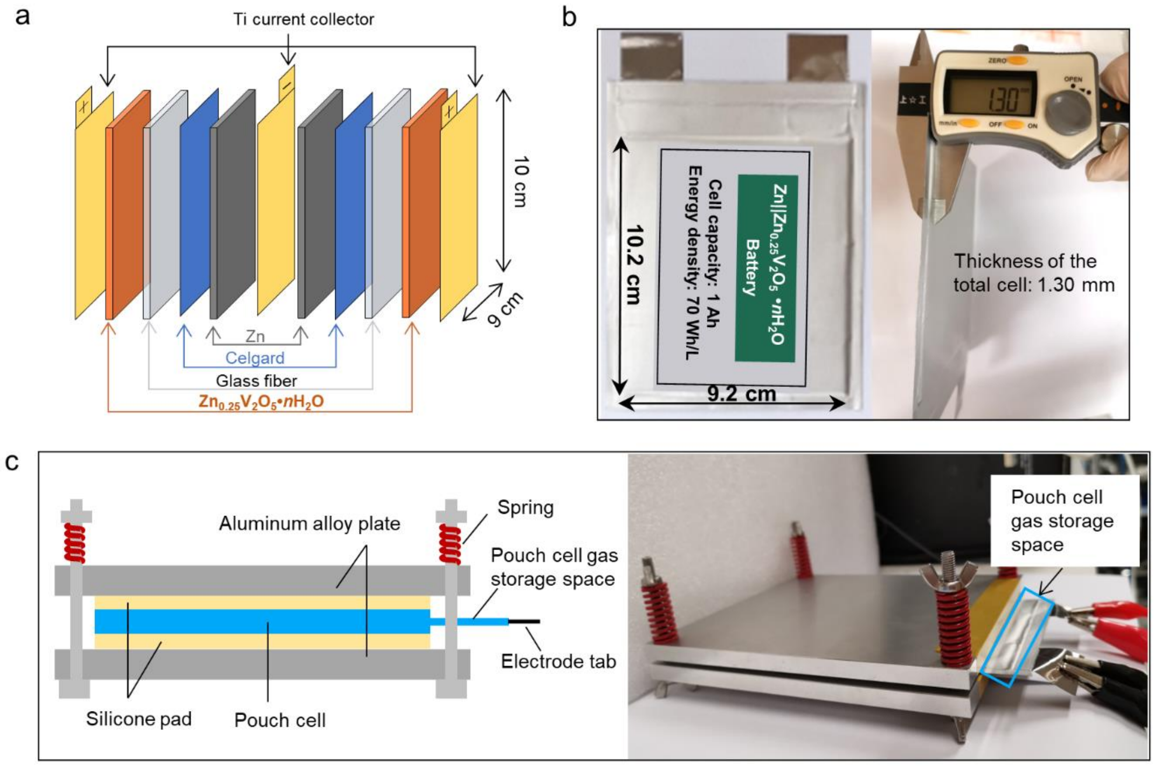

Supplementary Fig. 29 | The structure and photos of the pouch cell. a, The schematic of the bipolar structure of the pouch cell. **b**, The photos and total thickness of the pouch cell. **c**, The schematic and photo of the testing plates. The testing plates were used to offer a uniform external pressure of 0.1 MPa.

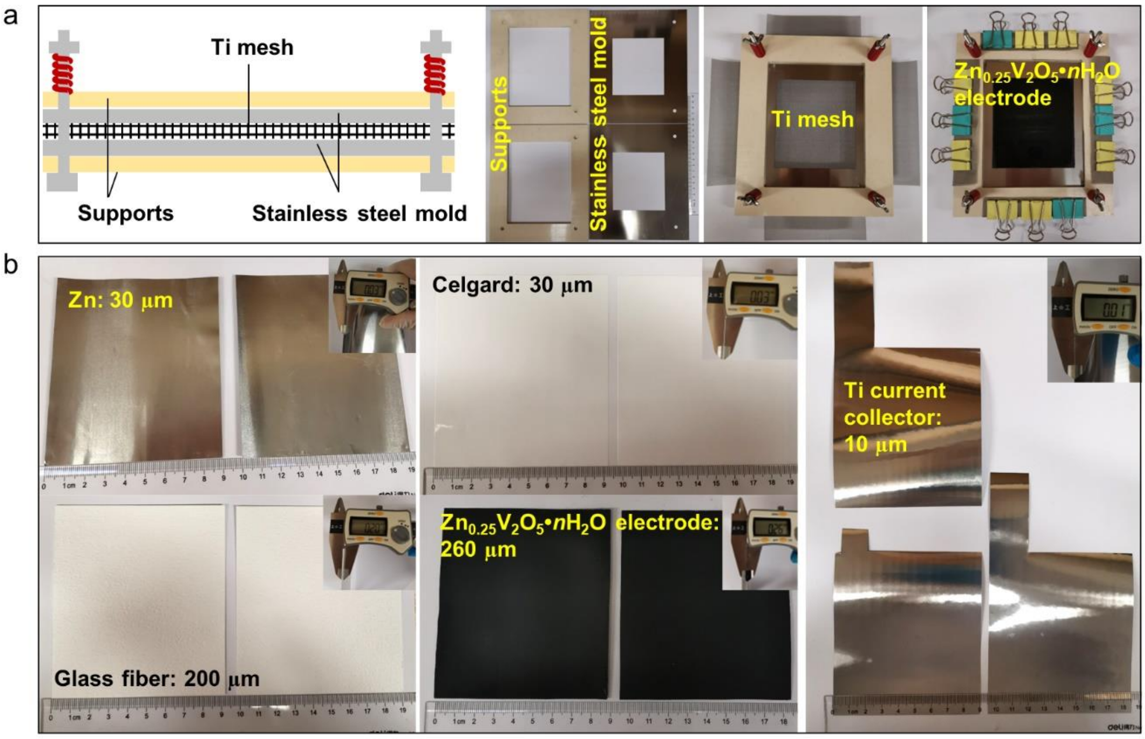

Supplementary Fig. 30 | The $Zn_{0.25}V_2O_5{\cdot}nH_2O$ positive electrode fabrication mode, photos of each component of the pouch cell. a, The structure and photos of the self-design positive electrode fabrication mode. **b**, The photos and size of each component in the pouch cell.

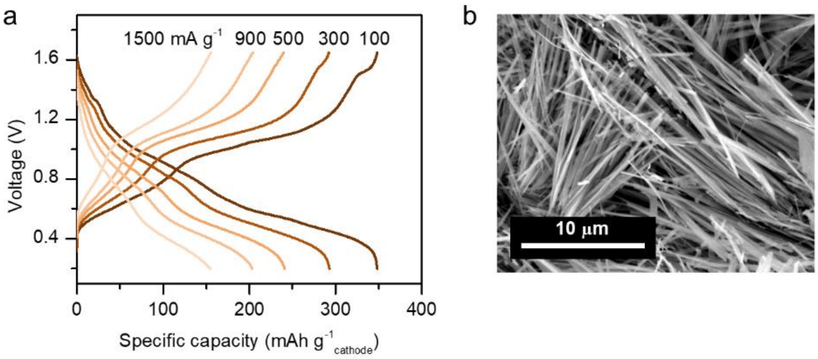

Supplementary Fig. 31 | The rate capability of Zn||$Zn_{0.25}V_2O_5{\cdot}nH_2O$ coin cell using the RME. a, The galvanostatic charging and discharging profiles of Zn||$Zn_{0.25}V_2O_5{\cdot}nH_2O$ coin cell using RME at different current density. The loading of the $Zn_{0.25}V_2O_5{\cdot}nH_2O$ was 8 mg cm^{-2}. Here the theoretical capacity of $Zn_{0.25}V_2O_5{\cdot}nH_2O$ is assumed to be 350 mAh g^{-1} (100 mA g^{-1}). **b**, The ex situ SEM image for the synthesized $Zn_{0.25}V_2O_5{\cdot}nH_2O$ nanobelts. The tests were conducted at ~25 ℃.

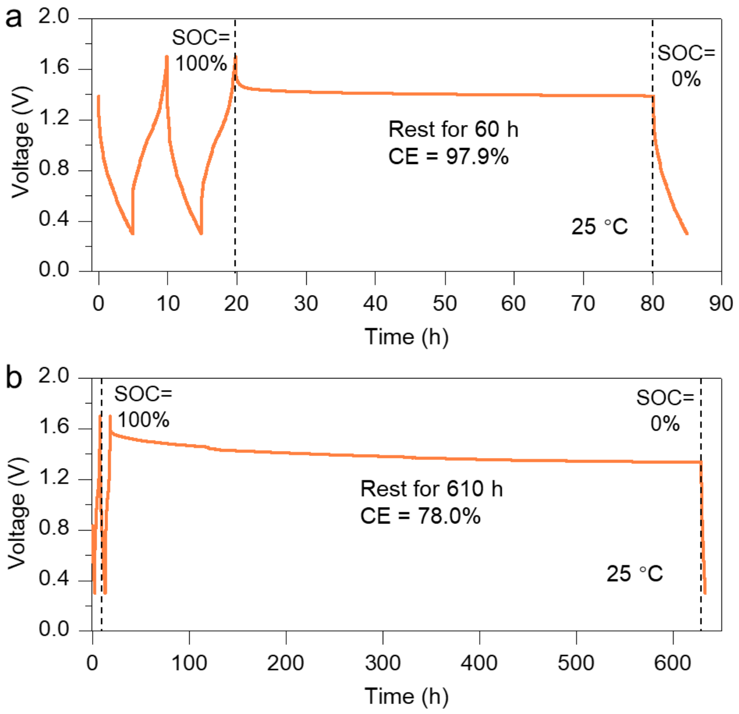

Supplementary Fig. 32 | The self-discharge test for the 1 Ah Zn||$Zn_{0.25}V_2O_5$·nH_2O pouch cell with N/P ratio of 1.07:1 using the RME at ~25 °C. The cell was assembled by coupling a Zn (10 µm, 5.9 mAh cm^{-2}) foil and a $Zn_{0.25}V_2O_5$·nH_2O (5.5 mAh cm^{-2}) electrode that was firstly processed by charging to 1.7 V to release the stored Zn^{2+}. The assembled pouch cell was firstly discharged and charged between 0.3 V and 1.7 V for two cycles and holding at OCV for 60 h (**a**) and 610 h (**b**) and then discharged to 0.3 V. The specific current is 56 mA $g^{-1}_{cathode}$. Other parameters of the cell components are same to Supplementary Fig. 29–30 and Supplementary Table 7.

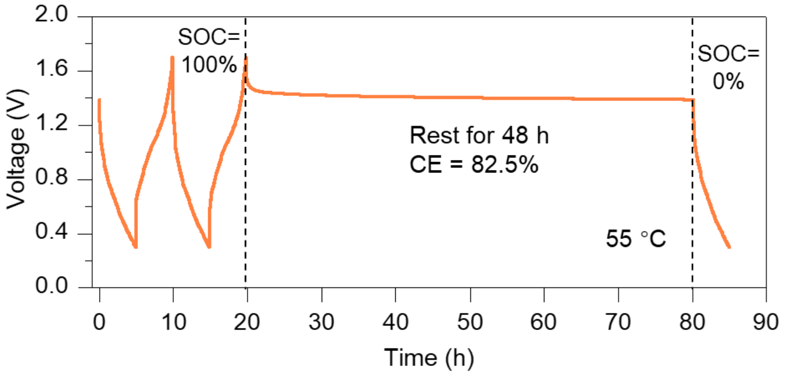

Supplementary Fig. 33 | The self-discharge test for the 1 Ah Zn||$Zn_{0.25}V_2O_5$·nH_2O pouch cell with N/P ratio of 1.07:1 using the RME at 55 °C. The cell was assembled by coupling a Zn (10 µm, 5.9 mAh cm^{-1}) foil and a $Zn_{0.25}V_2O_5$·nH_2O (5.5 mAh cm^{-1}) electrode that was firstly processed by charging to 1.7 V to release the stored Zn^{2+}. The assembled pouch cell was firstly discharged and charged between 0.3 V and 1.7 V for two cycles and holding at OCV for 48 h and then discharged to 0.3 V. The specific current is 56 mA $g^{-1}{}_{cathode}$. Other parameters of the cell components are same to Supplementary Fig. 29–30 and Supplementary Table 7.

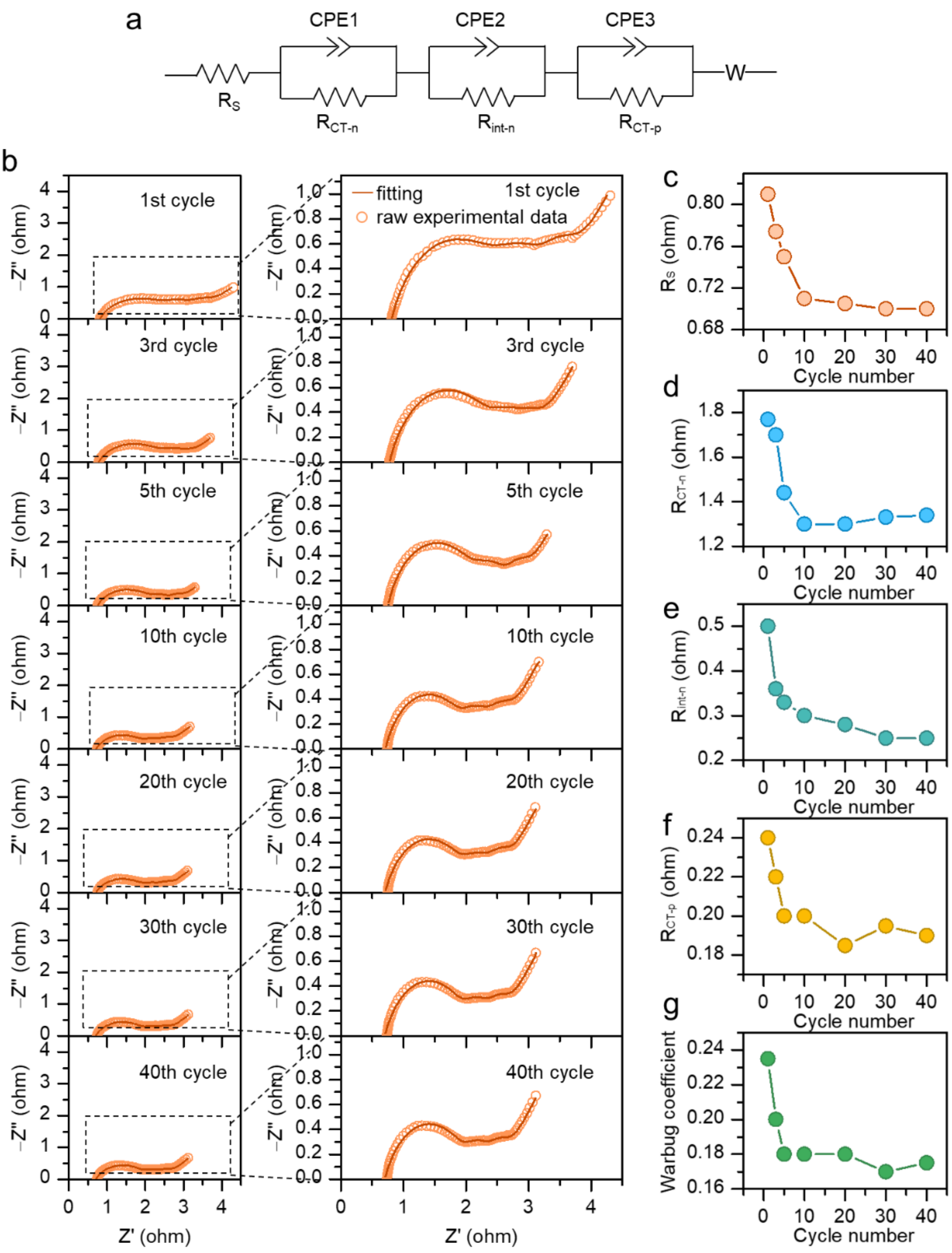

Supplementary Fig. 34 | The impedance of the Zn||$Zn_{0.25}V_2O_5$•nH_2O pouch cell at different cycles. a, The equivalent circuit model for data fitting. **b**, The original EIS data and fitting curves at different cycles. **c**, The ohmic resistance (R_S). **d**, The charge transfer resistance of the negative electrode (R_{CT-n}). **e**, The interphase resistance of the negative electrode (R_{int-n}). **f**, The charge transfer resistance of the positive electrode (R_{CT-p}). **g**, The Warburg coefficient. The interphase resistance of the negative electrode (R_{CT-n}) was contributed by the passivation layer (e.g. $Zn(OH)_2$) which was formed due to the initial side reaction between the Zn foil and the electrolyte. The decreasing of the impedances in the first 10 cycles can be due to the increased surface area of the Zn electrode under large areal capacity operation (5.5 mAh cm^{-2}) and the activation of the positive electrode. The tests were conducted at ~25 ℃.

Supplementary Table 1 | The fitted impedance results of Zn||Zn cell in various electrolytes.

Cycle number	R_S (ohm)	R_{CT} (ohm)	CPE1	R_{int} (ohm)	CPE2	Fitting error of $\chi/N^{0.5}$
H_2O	2.0	50	0.005	50	0.292	0.038
PAM	3.1	61	0.031	50	0.928	0.046
EG-H_2O	3.7	72.5	0.007	68	0.166	0.052
WiSE	3.5	105	0.004	80	0.134	0.067
RME	3.6	69	0.013	72	0.201	0.045

Supplementary Table 2 | The experimental database for evaluating the overall reversibility of Zn^0 anode in the five electrolytes.

Evaluation criteria	Data source	Original data for evaluating Zn^0 anode reversibility in the five electrolytes				
		H_2O	PAM	EG-H_2O	WiSE	RME
(i) H_2 coevolution suppression	Fig. 3g	1.1885	0.7684	0.4853	0.0220	0.0215
(ii) Dead Zn inhibition	Fig. 3h	0.5754	0.3396	0.3397	0.2923	0.2585
(iii) Dendrite growth suppression	Supplementary Fig. 23	1005	872	652	2350	8000
(iv) Corrosion resistance	Supplementary Fig. 11a	435	388	190	93	150
(v) Reaction kinetics	Supplementary Fig. 24b	1.14×10^{-3}	7.80×10^{-4}	3.10×10^{-4}	9.70×10^{-5}	2.97×10^{-4}

Note, (i) the contribution of average H_2 coevolution (%) in Fig. 3g was used to evaluate the H_2 coevolution suppression; (ii) the contribution of average dead Zn (%) in Fig. 3h was used to evaluate the dead Zn inhibition; (iii) for the dendrite growth, the unit of the data is "h"; (iv) for the corrosion resistance, the unit of the data is "nmol"; (v) for the reaction kinetics, the unit of the original data is "A cm^{-2}".

Supplementary Table 3 | The reversibility grade database for evaluating the overall reversibility of Zn^0 anode in the five electrolytes.

Evaluation criteria	Zn^0 anode reversibility grade in the five electrolytes				
	H_2O	PAM	EG-H_2O	WiSE	RME
(i) H_2 coevolution suppression	0.8414	1.3014	2.0606	45.4545	46.5116
(ii) Dead Zn inhibition	1.7379	2.9446	2.9438	3.4211	3.8685
(iii) Dendrite growth suppression	1005	872	652	2350	8000
(iv) Corrosion resistance	0.0023	0.0026	0.0053	0.0107	0.0067
(v) Reaction kinetics	−2.9431	−3.1079	−3.5086	−4.0132	−3.5272

Note, the reversibility grades for the "H_2 coevolution suppression", "Dead Zn inhibition", and "Corrosion resistance" were the reciprocal of the original data in Supplementary Table 2; the reversibility grades for the "Reaction kinetics" were the logarithm of the original data in Supplementary Table 2.

Supplementary Table 4 | The normalized reversibility grade for evaluating the overall reversibility of Zn^0 anode in the five electrolytes in Fig. 4a−4e.

Evaluation criteria	Normalized Zn^0 anode reversibility grade in the five electrolytes				
	H_2O	PAM	EG-H_2O	WiSE	RME
(i) H_2 coevolution suppression	0	0.04029	0.1068	3.9074	4
(ii) Dead Zn inhibition	0	2.2652	2.2638	3.1598	4
(iii) Dendrite growth suppression	0.1922	0.1198	0	0.9243	4
(iv) Corrosion resistance	0	0.1429	1.4286	4	2.0952
(v) Reaction kinetics	4	3.3843	1.8864	0	1.8168

Supplementary Table 5 | The cell parameters for laboratory-scale coin cell in the literature and ampere-hour-scale pouch cell in this work (Fig. 5a).

	Negative electrode Zn (μm)	Negative electrode (mAh cm^{-2})	Positive electrode (mAh cm^{-2})	N/P ratio	Total discharge capacity (mAh)	Electrolyte (μL)	E/C ratio (g Ah^{-1})
Coin cell	100	58.8	0.7 (ref[3,4])	**84:1**	1.08 (Electrode area is 1.54 cm^{-2})	75 (ref[5])	**92**
Pouch cell (this work)	30	17.6	~5.5	**3.2:1**	1000 (Electrode area=90 cm^{-2}, 2 pieces)	7000	**9.3**

Note, the electrolyte density is assumed to be 1.33 g mL^{-1} (RME, ~25 °C); for the areal capacity of positive electrode in the coin cell, the specific capacity of active material is assumed to be 350 mAh g^{-1} (Supplementary Fig. 31), the loading mass is assumed to be 2 mg cm^{-2} (ref[3,4,6]), and the area of the positive electrode is assumed to be 1.54 cm^2 (the diameter of commonly used electrode is 14 mm).

Supplementary Table 6 | Energy calculations and technical specifications of the Zn||V_2O_5 coin cell with 3 M $Zn(CF_3SO_3)_2$ aqueous electrolyte solution.

Electrodes	Electrolyte	Specific energy (Wh/kg)	Electrode areal (cm^{-2})	Loading mass (mg cm^{-2})	Cell volume (L)	Energy density (Wh L^{-1})	Reference
Zn||V_2O_5	3 M $Zn(CF_3SO_3)_2$	322 (cathode)	1.54 (a)	2	0.72*10^{-3} (b)	1.38	ref[7]

Note, (a) the diameter of the electrode is assumed to be 14 mm (CR2032); (b) the cell volume is inner volume of the coin cell and calculated as 3.14×0.845×0.845×0.32×10^{-3} (L) (the diameter of the negative electrode shell is 16.9 mm); (c) the specific energy is calculated based on the mass of the active material in the cathode.

Supplementary Table 7 | The parameters of the pouch cell components.

Pouch cell components	Length (mm)	Width (mm)	Thickness (mm)	Weight (g)
Negative electrode (Zn foil)	100	90	0.03×2 pieces	1.92×2 pieces
Positive electrode ($Zn_{0.25}V_2O_5$•nH_2O and Ti mesh)	100	90	0.26×2 pieces	3.3×2 pieces
Separator (Glass fiber)	102	92	0.20×2 pieces	0.4×2 pieces
Separator (Celgard)	102	92	0.03×2 pieces	0.5×2 pieces
Current collector (Ti foil)	100	90	0.01×3 pieces	~0.4×3 pieces
Electrolyte (RME)	–	–	–	9.3 (7 mL)

Supplementary Table 8 | Comparison of the RME Zn metal pouch cell with the state-of-the-art literature of Zn metal pouch cells.

Electrodes	Electrolyte	Zn utilization	Areal capacity (mAh cm^{-2})	Total capacity (mAh)	Cycle number	Life time (h)	Reference
Zn||MnO_2	3 M $Zn(OTf)_2$ + 0.1 M $Mn(OTf)_2$	—	—	1550	50	223	ref[8]
NGO@Zn||$LiMn_2O_4$	2 M Li_2SO_4 + 1 M $ZnSO_4$	36%	2.0	164	178 (81.5%)	< 356	ref[9]
Zn||$VOPO_4$	4 m $Zn(OTf)_2$ +0.5m $Me_3EtNOTf$	100%	~0.5	50	90 (80%)	< 90	ref[10]
Zn||PANI	2 M $ZnSO_4$ - 50%Methanol -50%H_2O (vol%)	—	~1	12	300 (~95.6%)	< 72	ref[11]
Zn||$Zn_{0.25}V_2O_5$•nH_2O	RME	31.2%	**5.5**	**1000**	390 (80%)	**3600**	This work

Note, for the Zn||PANI[11], the total area of the pouch cell was re-estimated as 3 cm × 4 cm; the values listed in "Life time (h)" column was estimated by "the time of the first cycle × cycle number" according to the reported values in the literature.

Supplementary Table 9 | The fitted impedance results of Zn||$Zn_{0.25}V_2O_5$•nH_2O pouch cell.

Cycle number	R_S (ohm)	R_{CT-n} (ohm)	R_{int-n} (ohm)	R_{CT-p} (ohm)	Warburg coefficient	Fitting error of $\chi/N^{0.5}$
1st	0.81	1.77	0.5	0.24	0.235	0.085
3rd	0.774	1.7	0.36	0.22	0.2	0.094
5th	0.75	1.44	0.33	0.2	0.18	0.076
10th	0.71	1.3	0.3	0.2	0.18	0.072
20th	0.705	1.3	0.28	0.185	0.18	0.072
30th	0.7	1.33	0.25	0.195	0.17	0.069
40th	0.7	1.34	0.25	0.19	0.175	0.065

Supplementary Note 1 | The stability of Zn^0 plating/stripping.

The stability of Zn^0 deposition/stripping and morphology of the deposited Zn^0 surface in the RME were examined via a Zn||Zn symmetric cell and scanning electron microcopy (SEM). As shown in Supplementary Fig. 3a, the Zn||Zn symmetric cell in the RME electrolyte delivered a long cycling for more than 1,100 h at 5 mA cm^{-2} and 5 mAh cm^{-2}. In sharp contrast, the cell in the 3 m $Zn(OTf)_2/H_2O$ (H_2O) electrolyte only survived for ~30 h before short circuit occurred. Meanwhile, substantial flake-like morphology was observed in the H_2O electrolyte (Supplementary Fig. 3b) in comparison with the relatively smooth surface and compact structure observed in the RME (Supplementary Fig. 3c). The uniform Zn^0 plating morphology can be attributed to the change of solvation environment of Zn^{2+} in the presence of sulfolane. As shown in Supplementary Fig. 4, the DFT results indicate that Zn^{2+} is highly tended to be solvated by sulfolane compared with water since the formation energy of $Zn^{2+}(sulfolane)_n(H_2O)_{6-n}$ (n=6, 5, 4, 2) complex is lower than that of $Zn^{2+}(H_2O)_6$. In the RME (H_2O:sulfolane = 16:4), five water molecules in the original primary solvation shell of Zn^{2+} were replaced by sulfulane leading to the formation of $Zn^{2+}(sulfolane)_5(H_2O)_1$ complex as shown in Supplementary Fig. 3e and Supplementary Fig. 5a−5c. The more stable solvation structure of Zn^{2+} in the RME implies that more energy thus higher overpotential is required for Zn^0 deposition which enables the kinetics for homogeneous Zn^0 deposition[12-14].

Supplementary Note 2 | The formation and recovery of isolated Zn.

As shown in Supplementary Fig. 19, upon Zn^0 plating in early cycles, dendritic morphology evolves due to the uneven deposition of Zn metal. In the stripping process, due to the nonuniform dissolution of Zn dendrites, some tips of the dendrites lose electric contact with the current collector leaving isolated Zn in the electrolyte adjacent to the electrode surface.

With this process repeated for many times, large amount of isolated Zn will accumulate and distribute randomly near the electrode surface. And in some stripping process, the isolated Zn (metallic state, Zn^0) was electrically reconnected with the current collector due to the polarization response to the electrical field of the electrolyte[15]. As the Zn on the substrate gradually stripped, the isolated Zn that reconnected with the current collector was dissolved and contributed extra capacity (Q_{is}) to the stripping capacity (Q_s). Since the isolated Zn was randomly distributed neighboring the electrode surface, the recovery thus the contribution of the isolated Zn (Q_{is}) is not stable for each cycle.

We further examine the polarization response of the isolated Zn to the electrical field of the electrolyte via an in situ optical microscope. As shown in Supplementary Fig. 20, the in situ optical microscope cell was constructed with Zn foil as anode, $Zn_{0.25}V_2O_5{\cdot}nH_2O$ as cathode, 3 m $Zn(OTf)_2/H_2O$ (H_2O) as electrolyte, and a Cu foil deposited with Zn metal (5 mAh cm^{-2}) mimicking the isolated Zn (labelled as Zn island). The $Zn_{0.25}V_2O_5{\cdot}nH_2O$ was used to provide a higher electric field intensity in the electrolyte when coupling with Zn metal anode for better observing the morphology change of Zn island. As shown in Supplementary Fig. 21, with the discharge proceeding to 30 min, and 60 min, obvious amorphous structure evolves and gradually grows up on the edge of the Zn-island that close to the Zn anode. This spatial progression can be attributed to the Zn^0 deposition due to the polarization response of the Zn-island to the electric field of the electrolyte[15]. Upon discharge, the electric field in the electrolyte points from the negative electrode (Zn foil) to the positive electrode ($Zn_{0.25}V_2O_5{\cdot}nH_2O$) (Supplementary Fig. 21a) where the Zn^{2+} moves from the negative electrode to the positive electrode[15]. Due to the polarization response to this electric field, the overpotential on the edge of the Zn-island near the Zn foil turns negative, causing Zn^{2+} in the neighborhood electrolyte reduced and deposited on the Zn-island. This leads to a gradual spatial progression of the Zn-island. And this spatial progression will eventually lead to the electric connection of Zn-island to Zn foil if the Zn-island is close enough to the Zn foil. This explains the recovery of isolated Zn in the Zn||Cu asymmetric cell (Supplementary Fig. 19). The formation and recovery of isolated Zn commonly occur in electrolytes where the dendrite growth is disastrous for example, the H_2O electrolyte.

Supplementary Note 3 | Calculation procedure of the normalized grades for Zn^0 anode reversibility in the five electrolytes as shown in Fig. 4a−4e.

Supplementary Table 2 lists the original database for evaluating the overall reversibility of Zn^0 anode in the five electrolytes. The reversibility grades in each criterion (Supplementary Table 3) are normalized to five grades with the lowest grade setting as "0" and highest grade setting as "4" to comprehensively compare the reversibility of Zn^0 anode in the five electrolytes. The increment of the five grades for each criterion was calculated by:

$$\text{Increment} = \frac{\text{highest grade in each critera} - \text{lowest grade in each critera}}{4}$$

For each criterion:

(i) H_2 coevolution suppression:

$$\text{Increment} = \frac{46.5116 - 0.8414}{4} = 11.4176$$

(ii) Dead Zn inhibition:

$$\text{Increment} = \frac{3.8685 - 1.7379}{4} = 0.5327$$

(iii) Dendrite growth suppression:

$$\text{Increment} = \frac{8000 - 652}{4} = 1837$$

(iv) Corrosion resistance:

$$\text{Increment} = \frac{0.0107 - 0.0023}{4} = 0.0021$$

(v) Reaction kinetics:

$$\text{Increment} = \frac{-2.9431 - (-4.0132)}{4} = 0.2675$$

Then the normalized grade for Zn^0 anode reversibility in each electrolyte (Supplementary Table 4) was calculated by:

$$\text{Normalized grade} = \frac{\text{reversibility grade in the electrolyte} - \text{lowest grade in corresponding criterion}}{\text{increment in corresponding criterion}}$$

The data of "reversibility grade in the electrolyte" and "lowest grade in corresponding criterion" are from Supplementary Table 3.

Supplementary Note 4 | The energy density of the pouch cell.

The energy density of the pouch cell was calculated based on the volume of all the components (Supplementary Table 7) except the Al soft packing films, the calculation procedure was as following:

$$\text{Volume} = \frac{102 \times 92 \times (0.03 \times 2 + 0.26 \times 2 + 0.20 \times 2 + 0.03 \times 2 + 0.01 \times 3)}{10^6} = 0.01\ \text{L}$$

$$\text{Energy density of the pouch cell} = \frac{0.7}{0.01} = 70\ \text{Wh L}^{-1}$$

Note that the electrolyte volume was not included here, as the spread of the electrolyte inside the cathode and separator pores leads to very little or no contribution to the volume of the overall stack[6]. This is supported by the similar values of the actual thickness of the inner materials and the calculated thickness of the inner materials (Supplementary Table 7), as following shows.

$$\text{Actual thickness of the inner materials} = 1.30 - 0.113 \times 2 = 1.074\ \text{mm}$$

$$\text{Caculated thickness of the inner materials} = (0.03 + 0.26 + 0.20 + 0.03) \times 2 + 0.01 \times 3 = 1.07\ \text{mm}$$

Here, the total thickness of the pouch cell is 1.30 mm (Supplementary Fig. 29b), and the thickness of the packing film is 113 µm.

Supplementary Note 5 | The specific energy of the pouch cell.

The specific energy of the pouch cell was calculated based on the weight of all the components

(Supplementary Table 7) except the Al soft packing films, the calculation procedure was as following:

$$\text{Specific energy (whole cell)} =$$

$$\frac{0.7}{((1.92 + 3.3 + 0.4 + 0.5) \times 2 + 0.4 \times 3 + 9.3) \times 10^{-3}} = 31 \text{ Wh kg}^{-1}$$

We further calculate the specific energy according to the weight of the active materials on the positive and negative materials:

$$\text{Specific energy (active materials)} = \frac{0.7}{(1.92 \times 2 + 3.6) \times 10^{-3}} = 94 \text{ Wh kg}^{-1}$$

Supplementary References

1. Zou, P. *et al.* Localized hydrophobicity in aqueous zinc electrolytes improves zinc metal reversibility. *Nano Lett.* **22**, 7535–7544 (2022).
2. Ma, L. *et al.* Realizing high zinc reversibility in rechargeable batteries. *Nat. Energy* **5**, 743-749 (2020).
3. Zampardi, G. & La Mantia, F. Open challenges and good experimental practices in the research field of aqueous Zn-ion batteries. *Nat. Commun.* **13**, 687 (2022).
4. Li, Y. *et al.* Understanding the gap between academic research and industrial requirements in rechargeable zinc-ion batteries. *Batteries Supercaps* **4**, 60-71 (2021).
5. Xiao, J. *et al.* Understanding and applying coulombic efficiency in lithium metal batteries. *Nat. Energy* **5**, 561-568 (2020).
6. Shang, Y. & Kundu, D. Understanding and performance of the zinc anode cycling in aqueous zinc-ion batteries and a roadmap for the future. *Batteries Supercaps* **5**, e202100394 (2022).
7. Zhang, N. *et al.* Rechargeable aqueous Zn–V_2O_5 battery with high energy density and long cycle life. *ACS Energy Lett.* **3**, 1366-1372 (2018).
8. Zhang, N. *et al.* Rechargeable aqueous zinc-manganese dioxide batteries with high energy and power densities. *Nat. Commun.* **8**, 405 (2017).
9. Zhou, J. *et al.* Ultrathin surface coating of nitrogen-doped graphene enables stable zinc anodes for aqueous zinc-ion batteries. *Adv. Mater.* **33**, 2101649 (2021).
10. Cao, L. *et al.* Fluorinated interphase enables reversible aqueous zinc battery chemistries. *Nat. Nanotechnol.* **16**, 902-910 (2021).
11. Hao, J. *et al.* Boosting zinc electrode reversibility in aqueous electrolytes by using low-cost antisolvents. *Angew. Chem., Int. Ed.* **60**, 7366-7375 (2021).
12. Qin, R. *et al.* Tuning Zn^{2+} coordination environment to suppress dendrite formation for high-performance Zn-ion batteries. *Nano Energy* **80**, 105478 (2021).
13. Han, D. *et al.* A non-flammable hydrous organic electrolyte for sustainable zinc batteries. *Nat. Sustain.* **5**, 205-213 (2022).
14. Yang, W. *et al.* Hydrated eutectic electrolytes with ligand-oriented solvation shells for long-cycling zinc-organic batteries. *Joule* **4**, 1557-1574 (2020).
15. Liu, F. *et al.* Dynamic spatial progression of isolated lithium during battery operations. *Nature* **600**, 659-663 (2021).
